# Supplementary figures and images for: The Distribution of the Asymptotic Number of Citations to Sets of Publications by a Researcher or from an Academic Department Are Consistent with a Discrete Lognormal Model
Source: PLoS One. 2015 Nov 16;10(11):e0143108. doi: 10.1371/journal.pone.0143108 (PMC4646658; doi:10.1371/journal.pone.0143108)

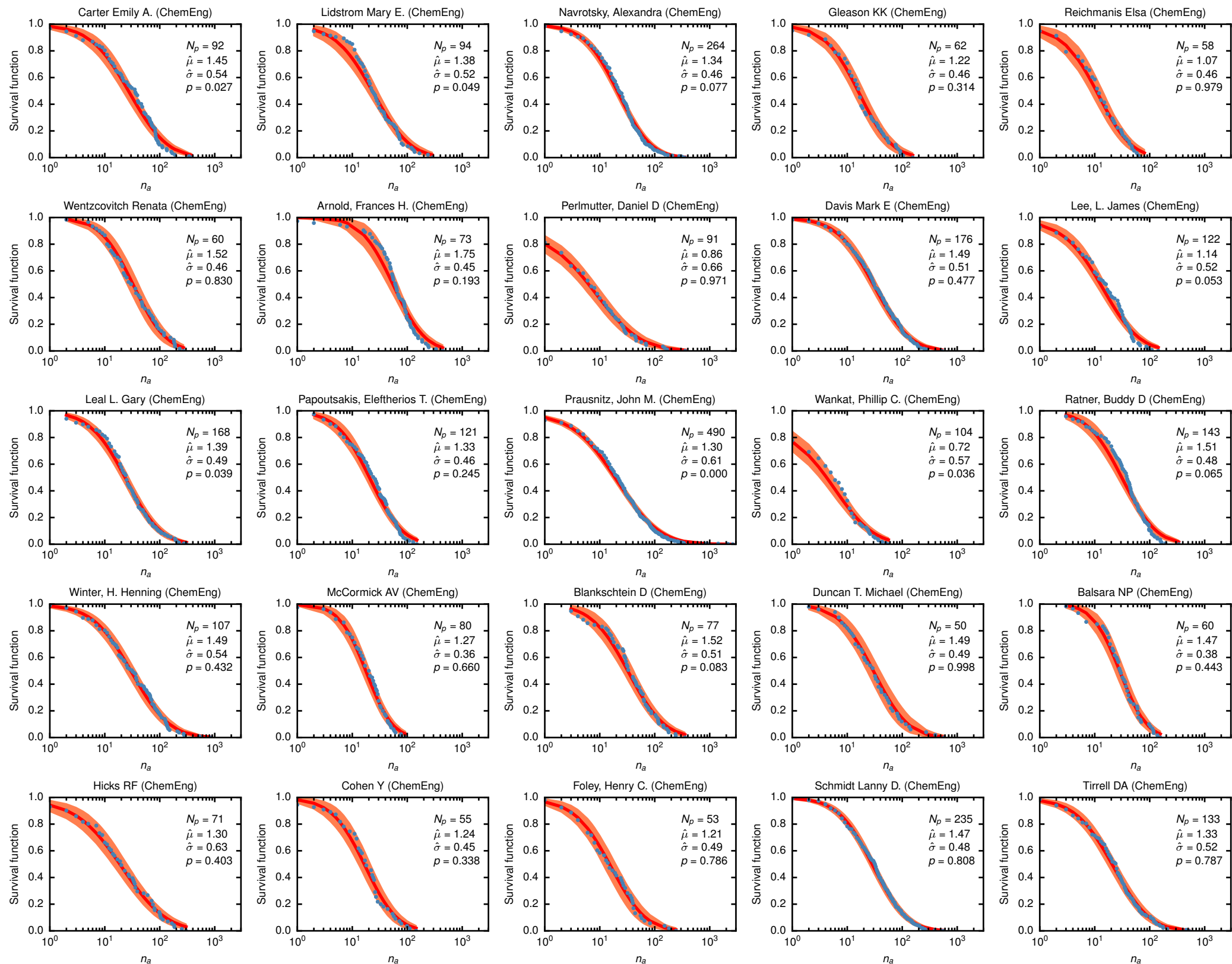

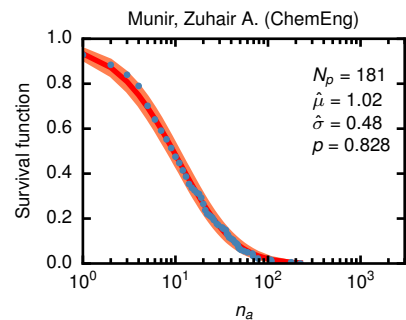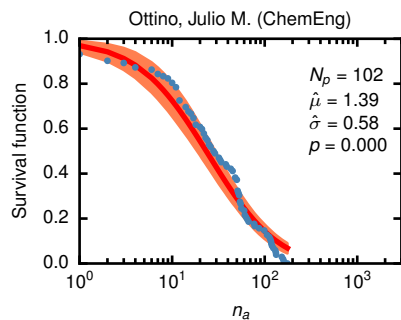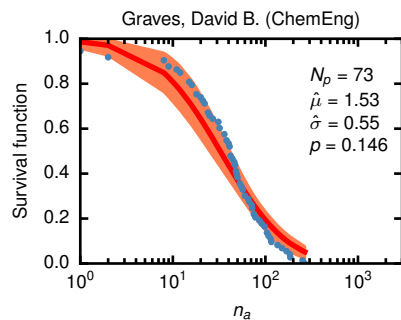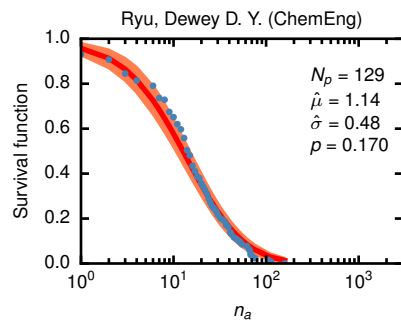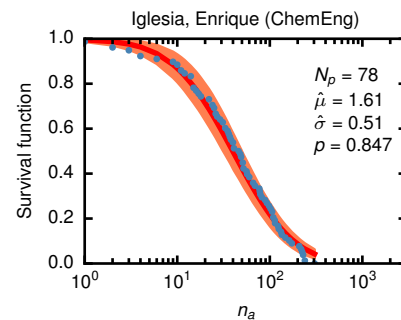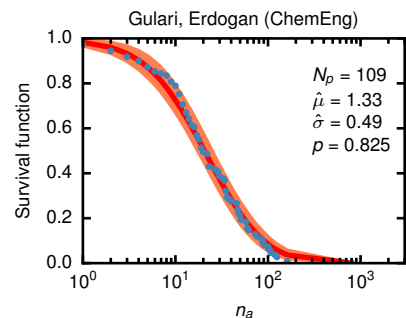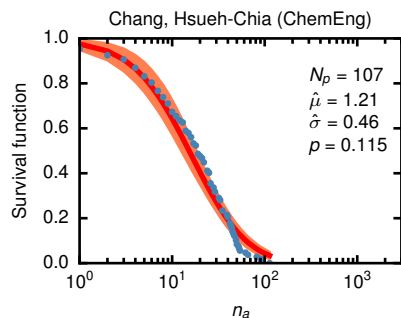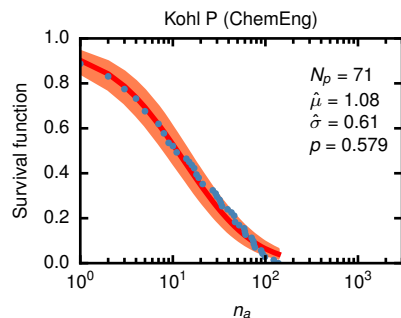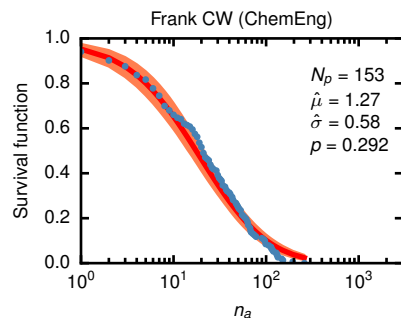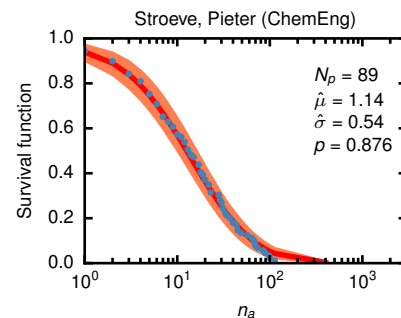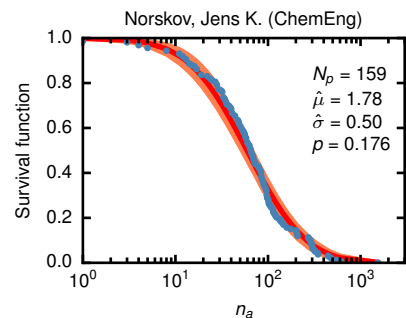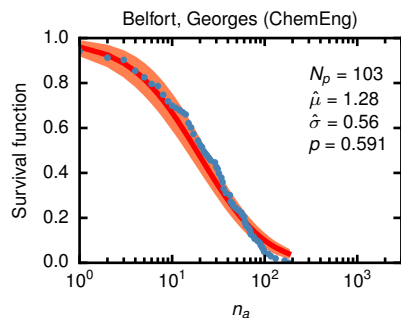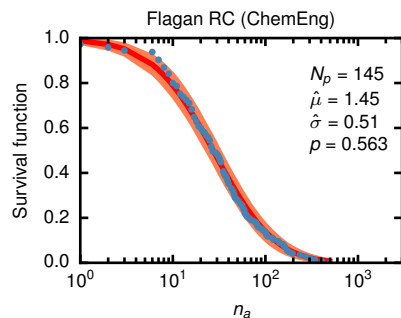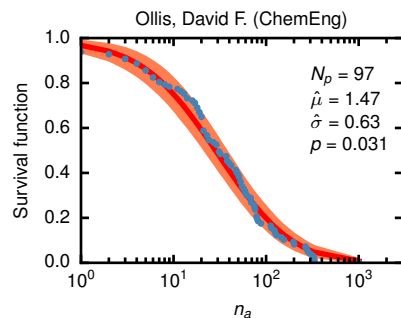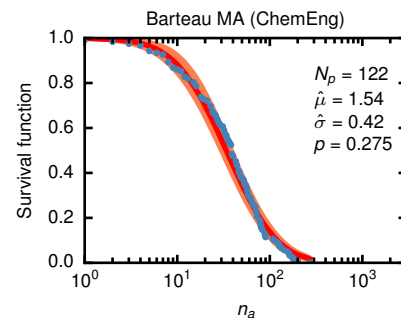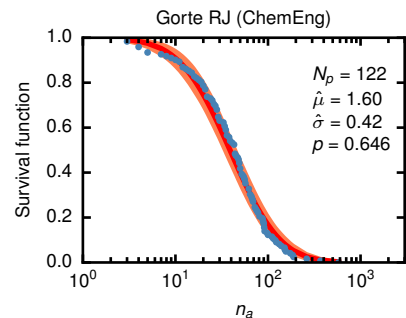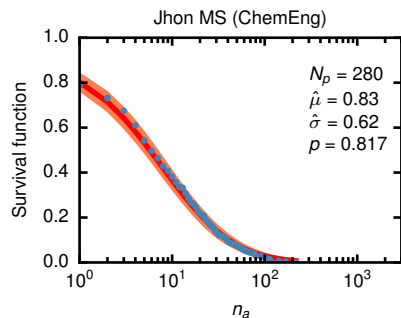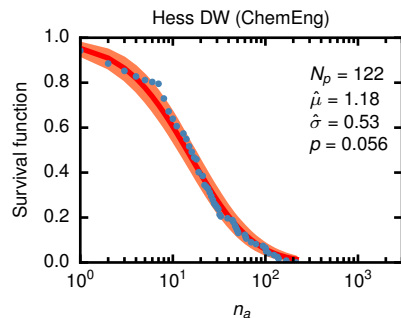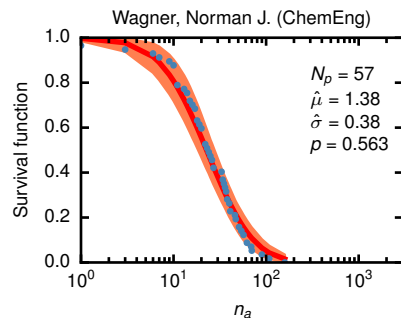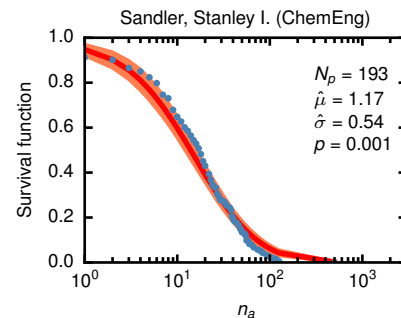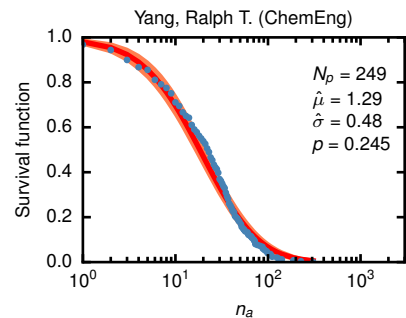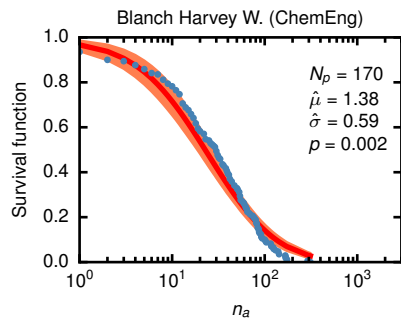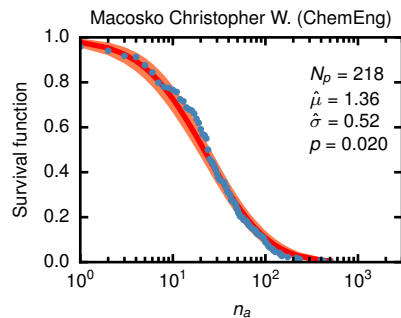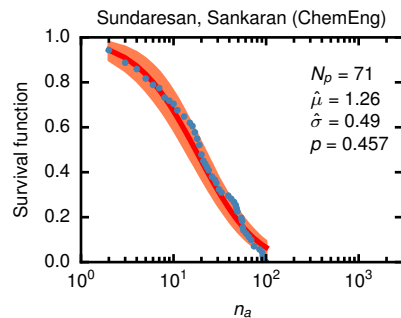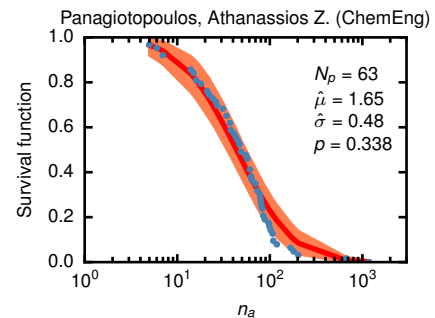

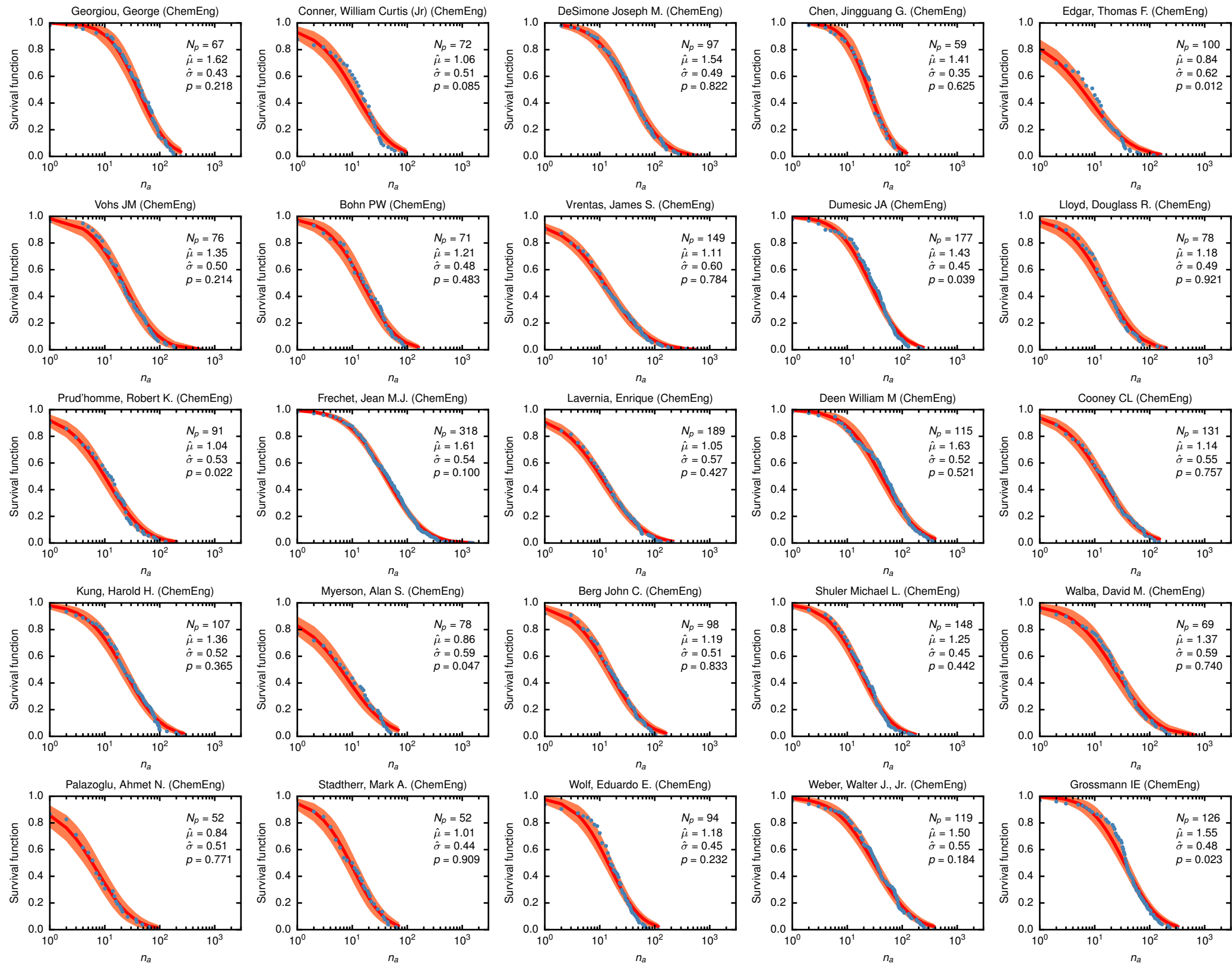

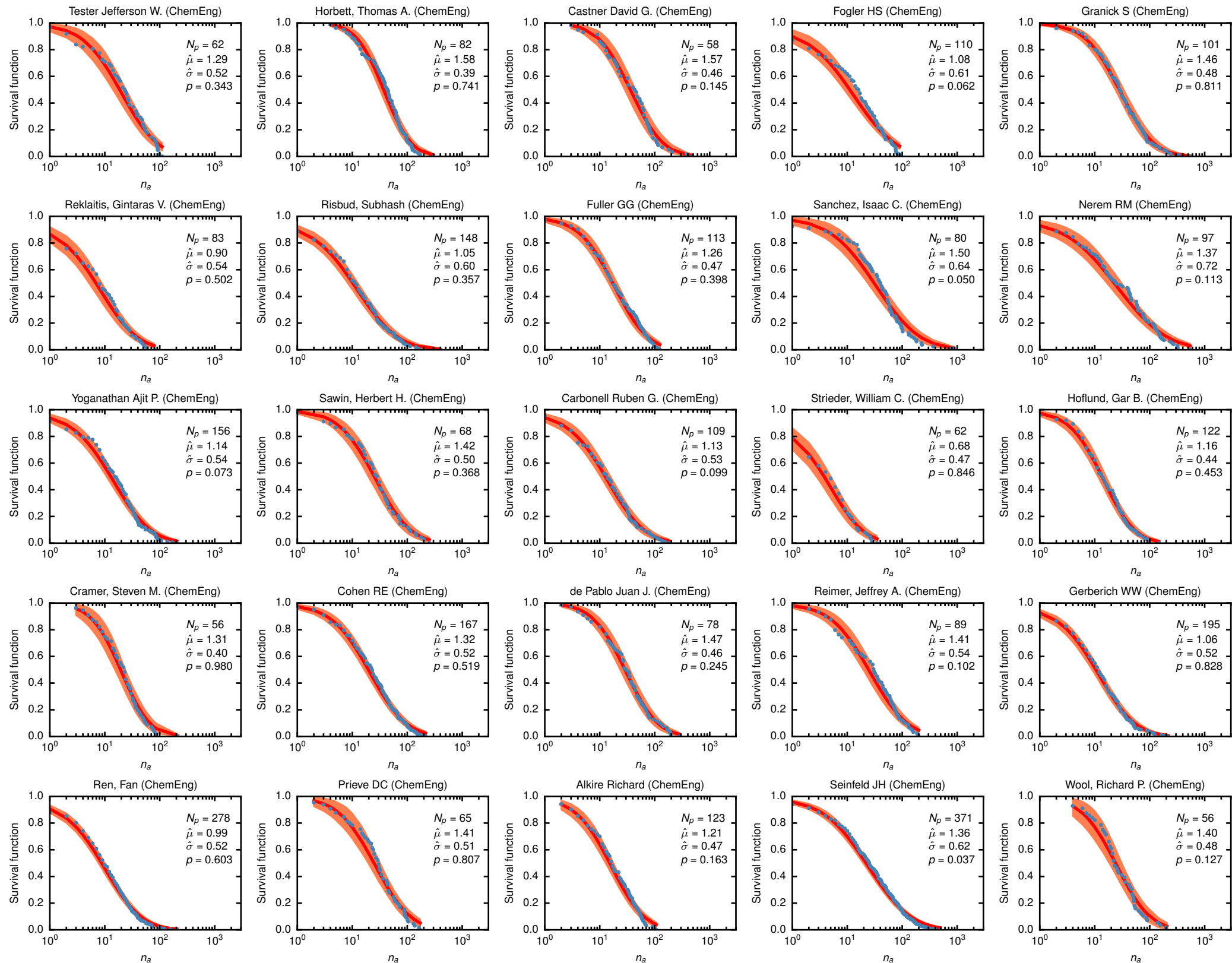

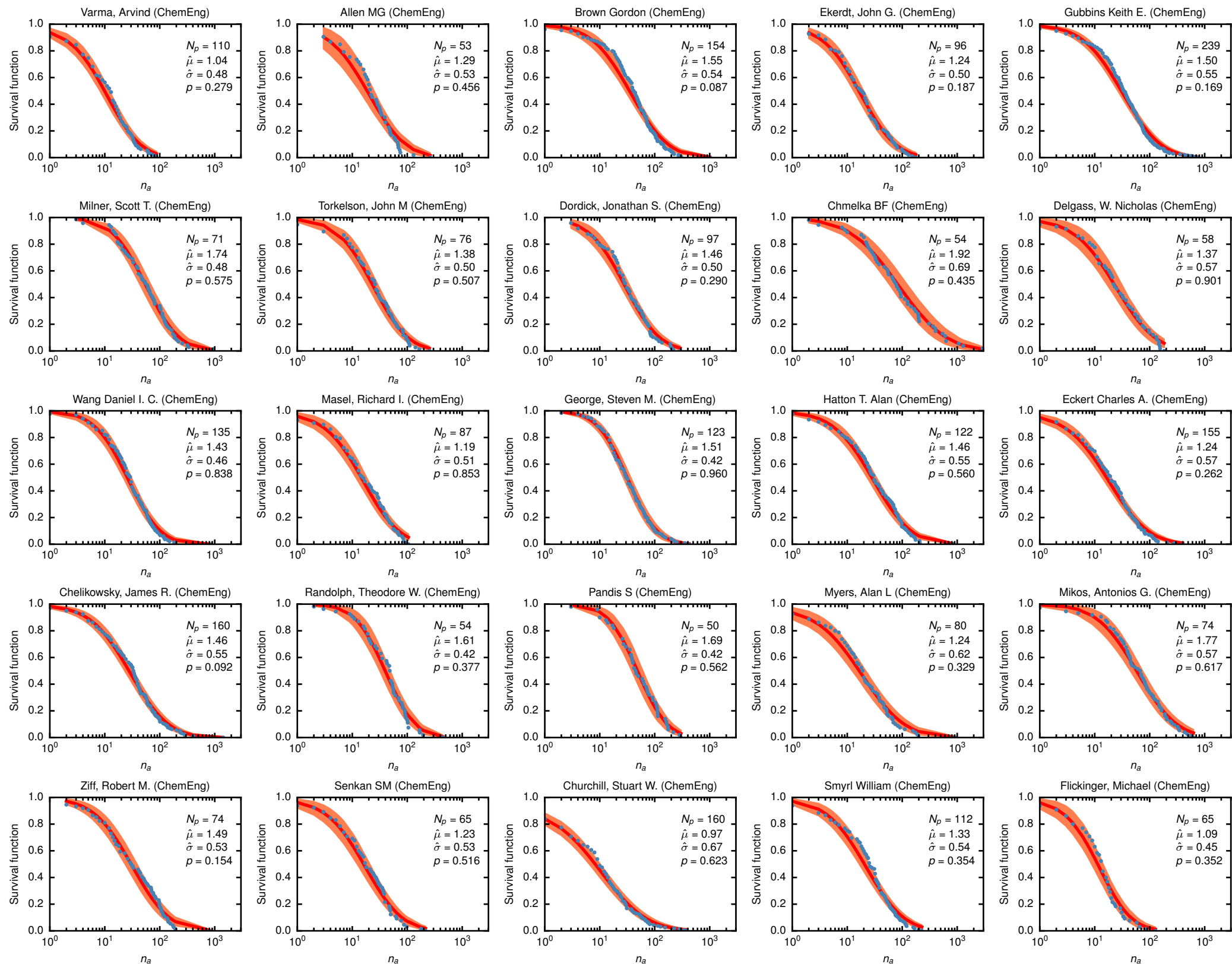

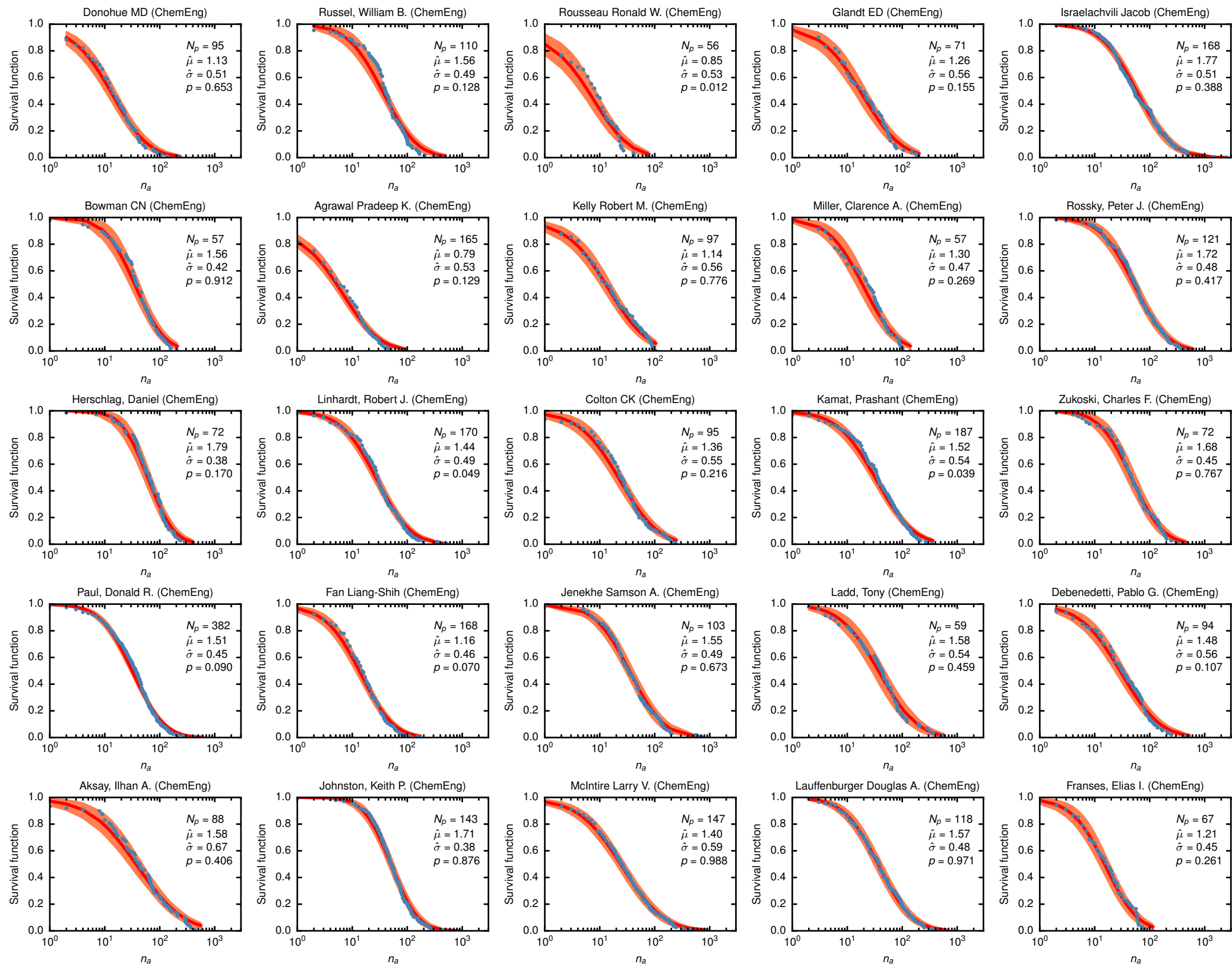

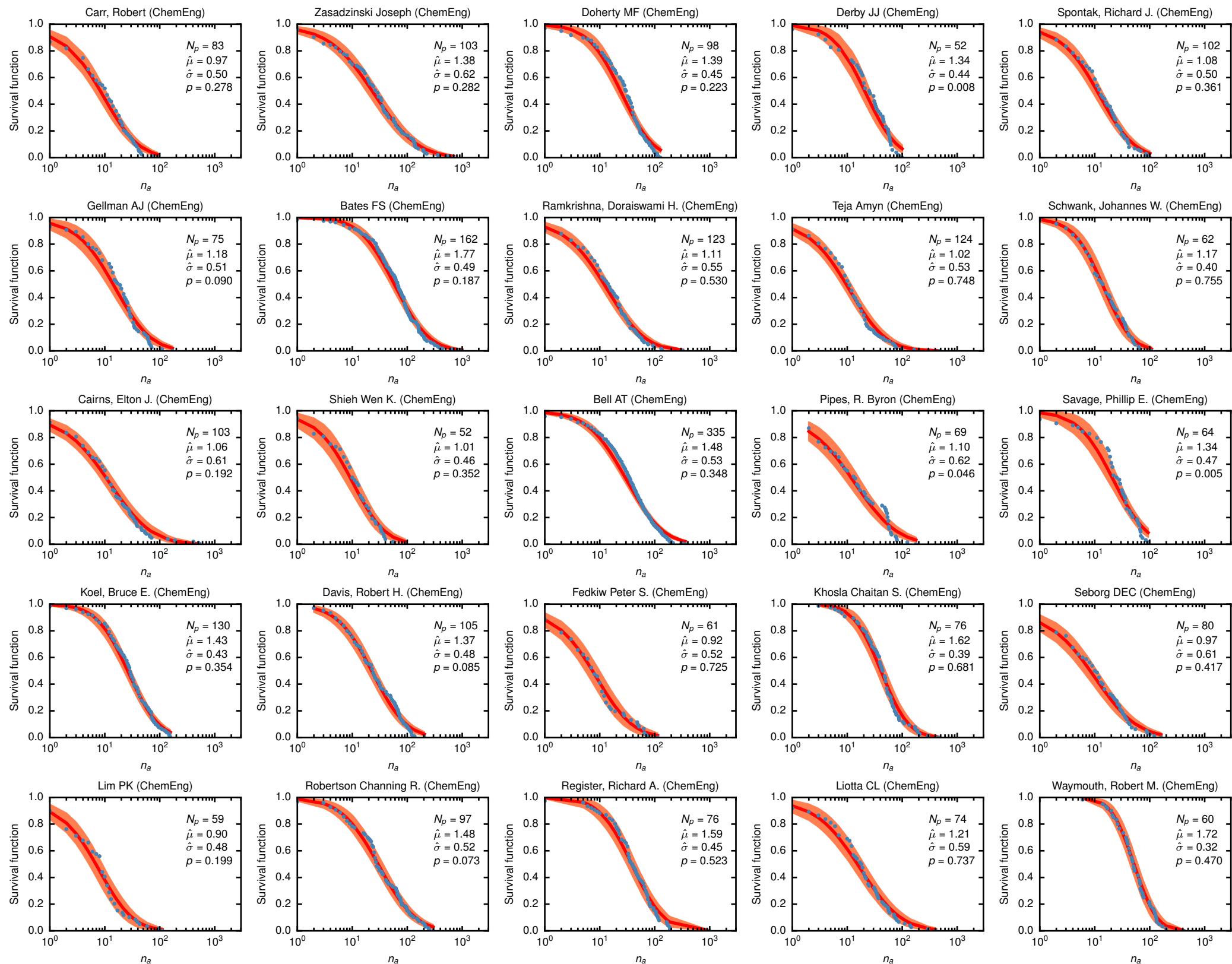

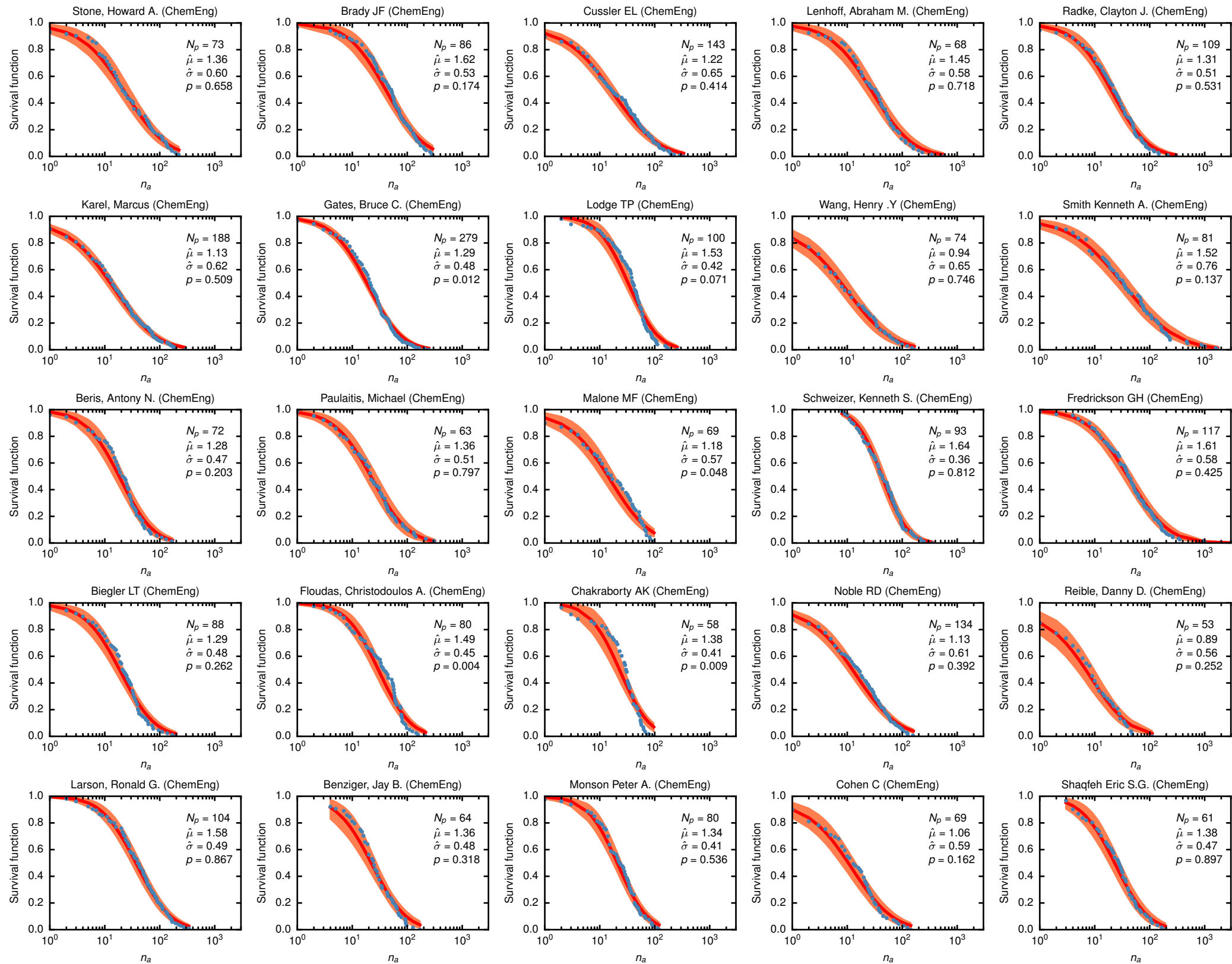

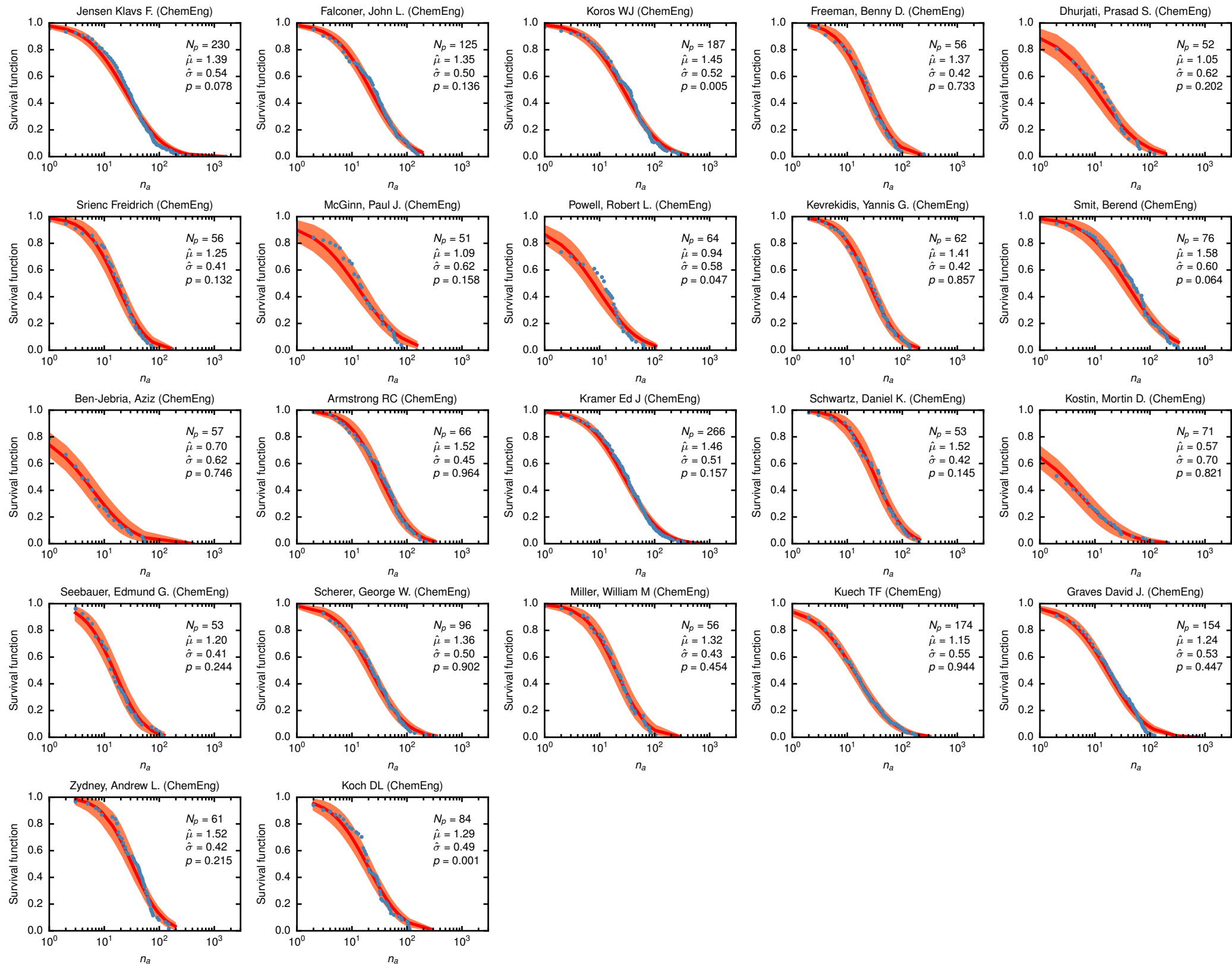

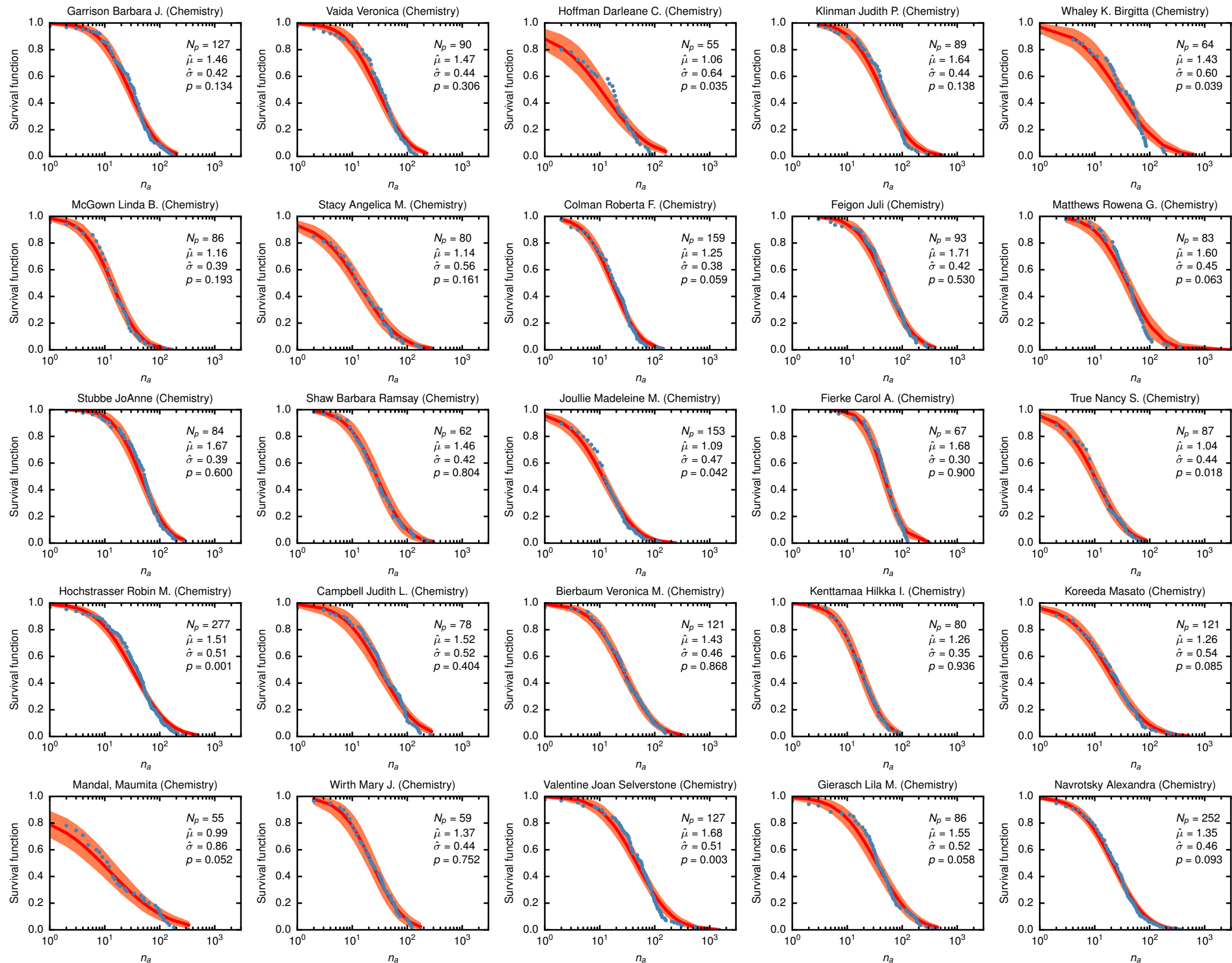



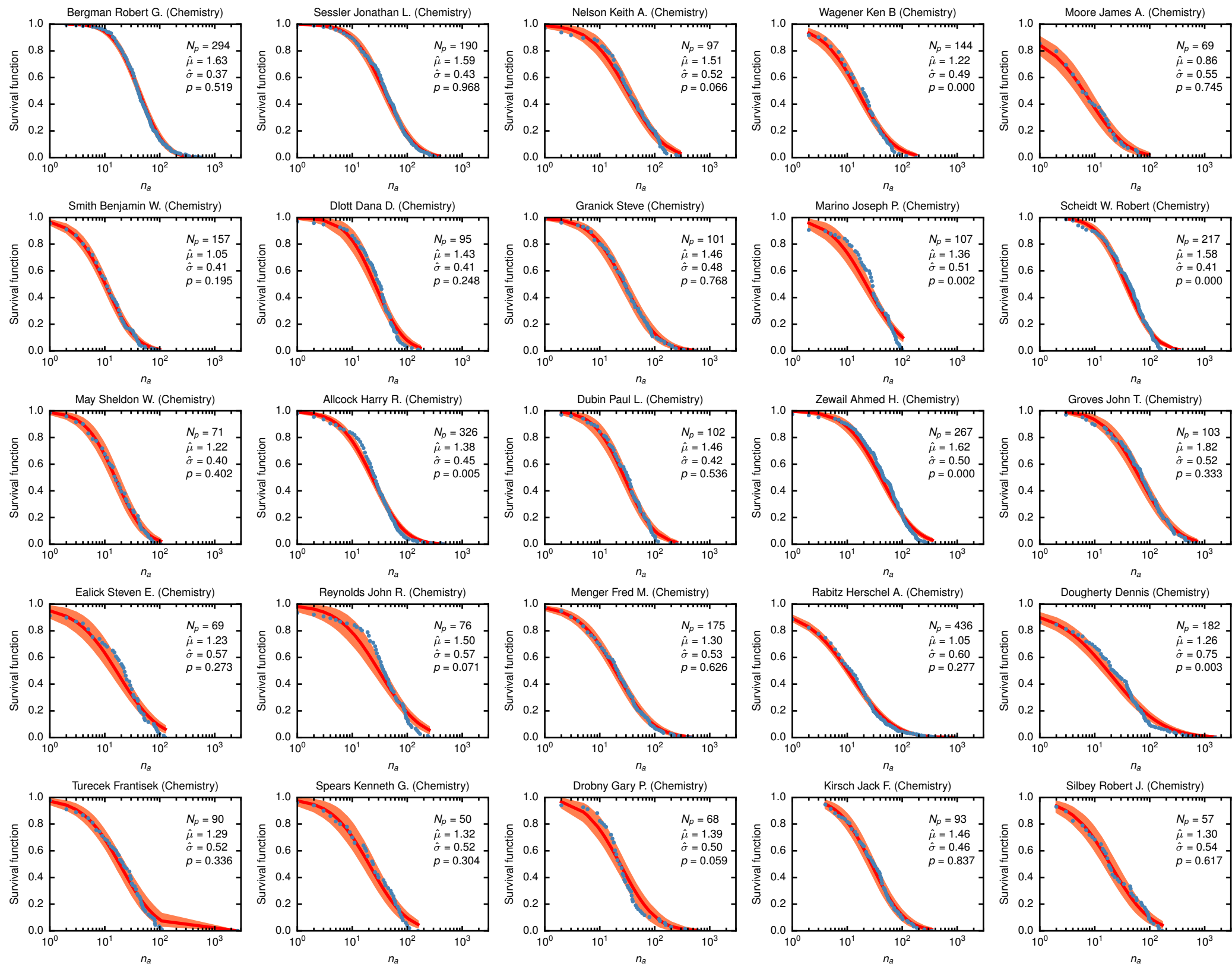

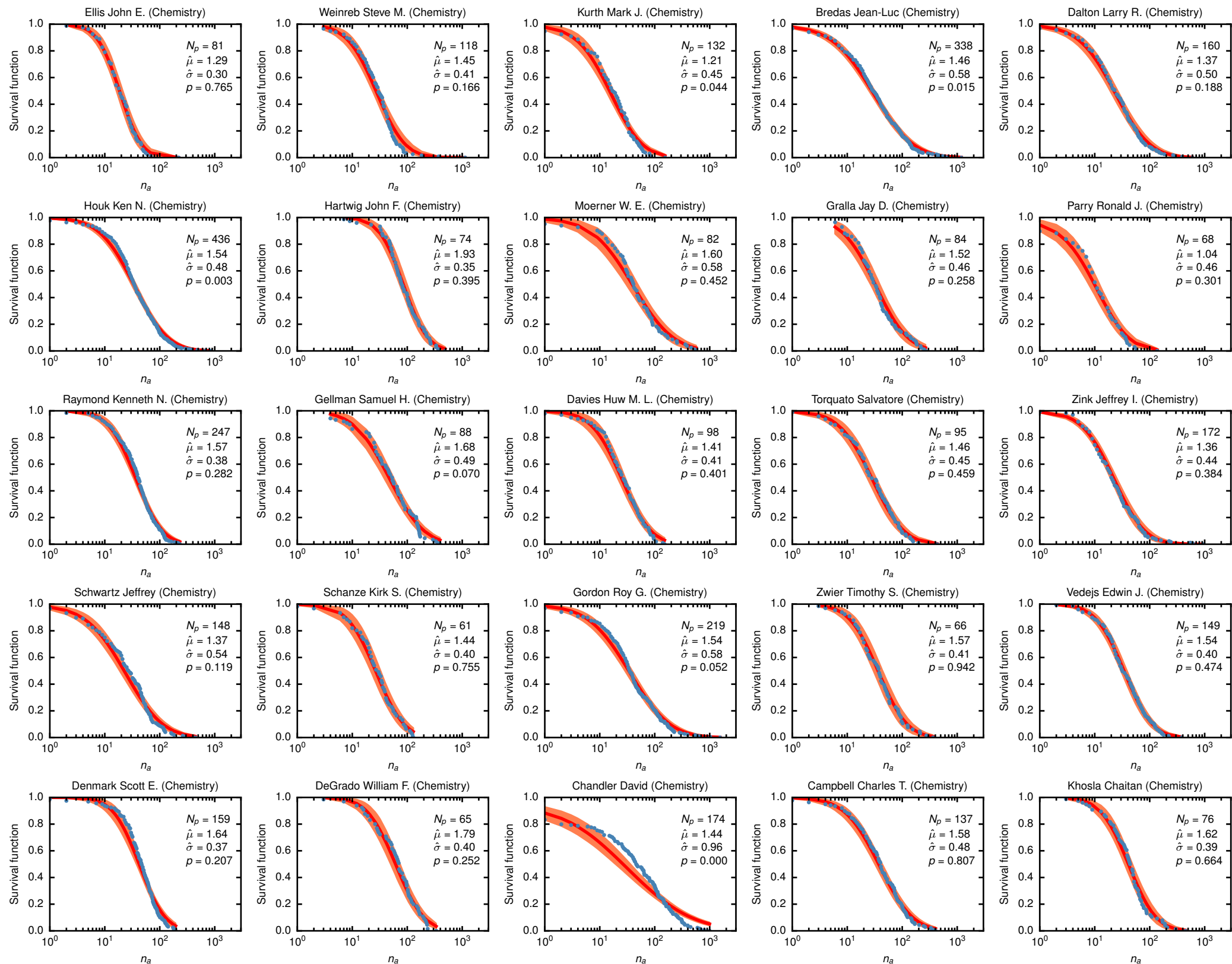

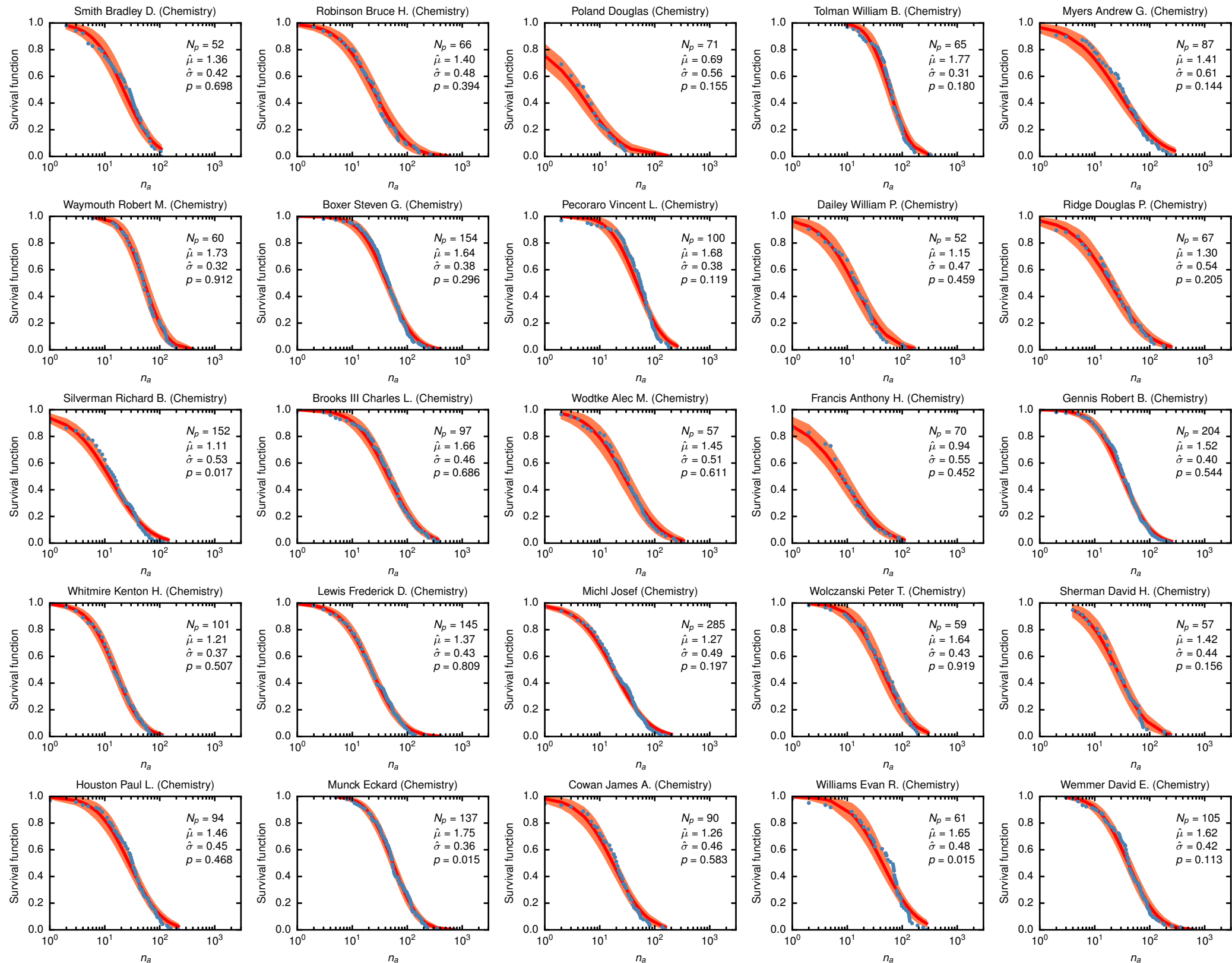



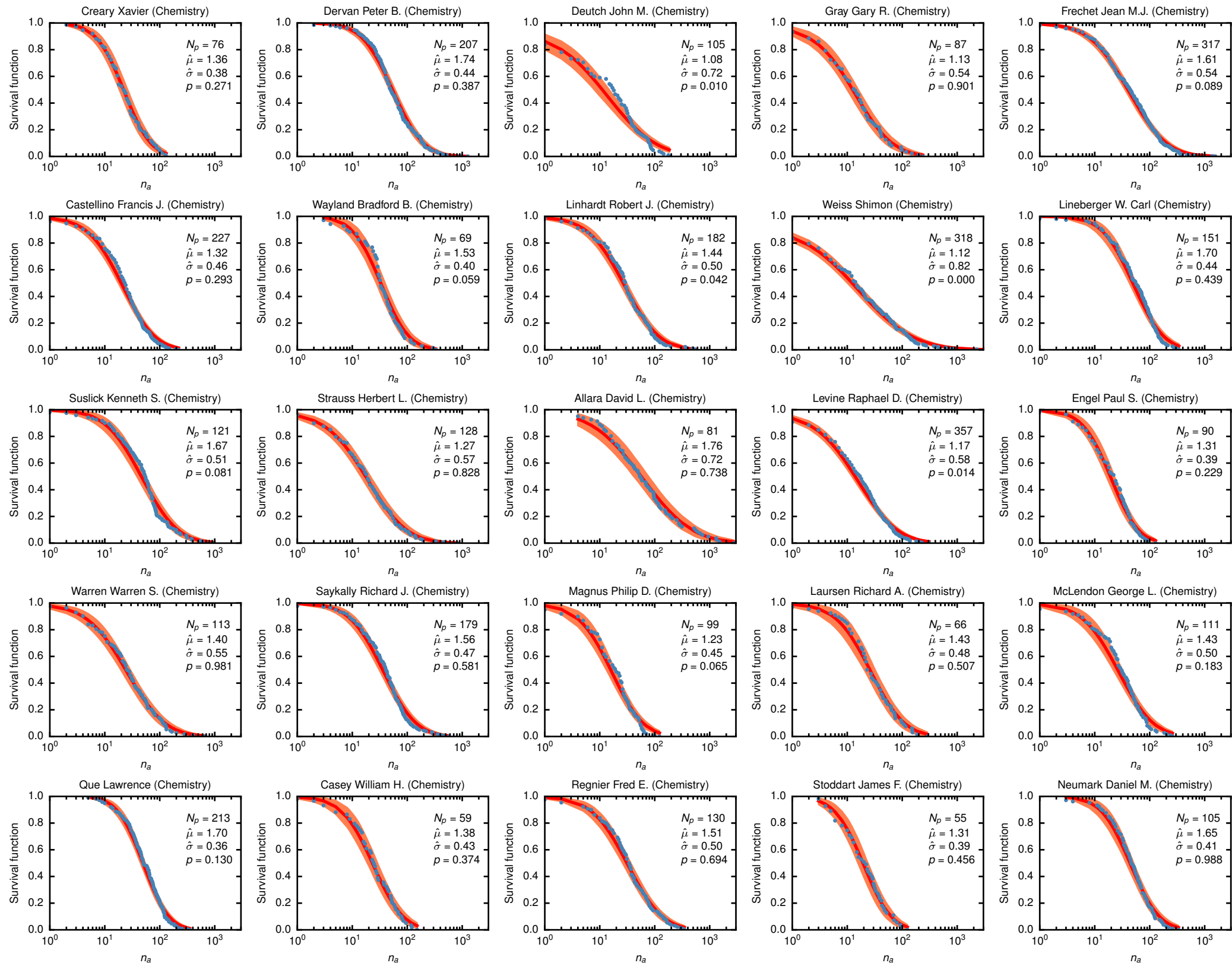

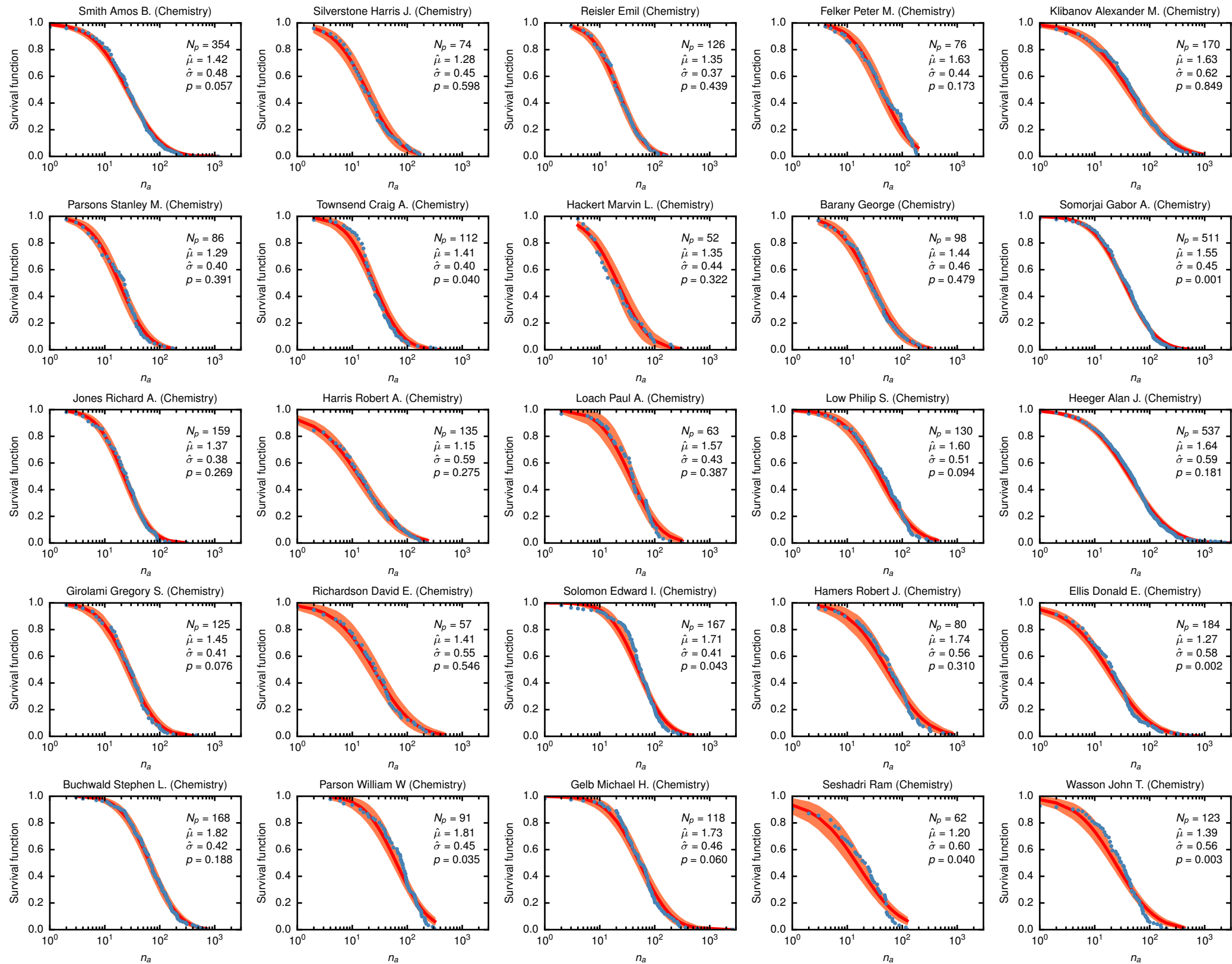

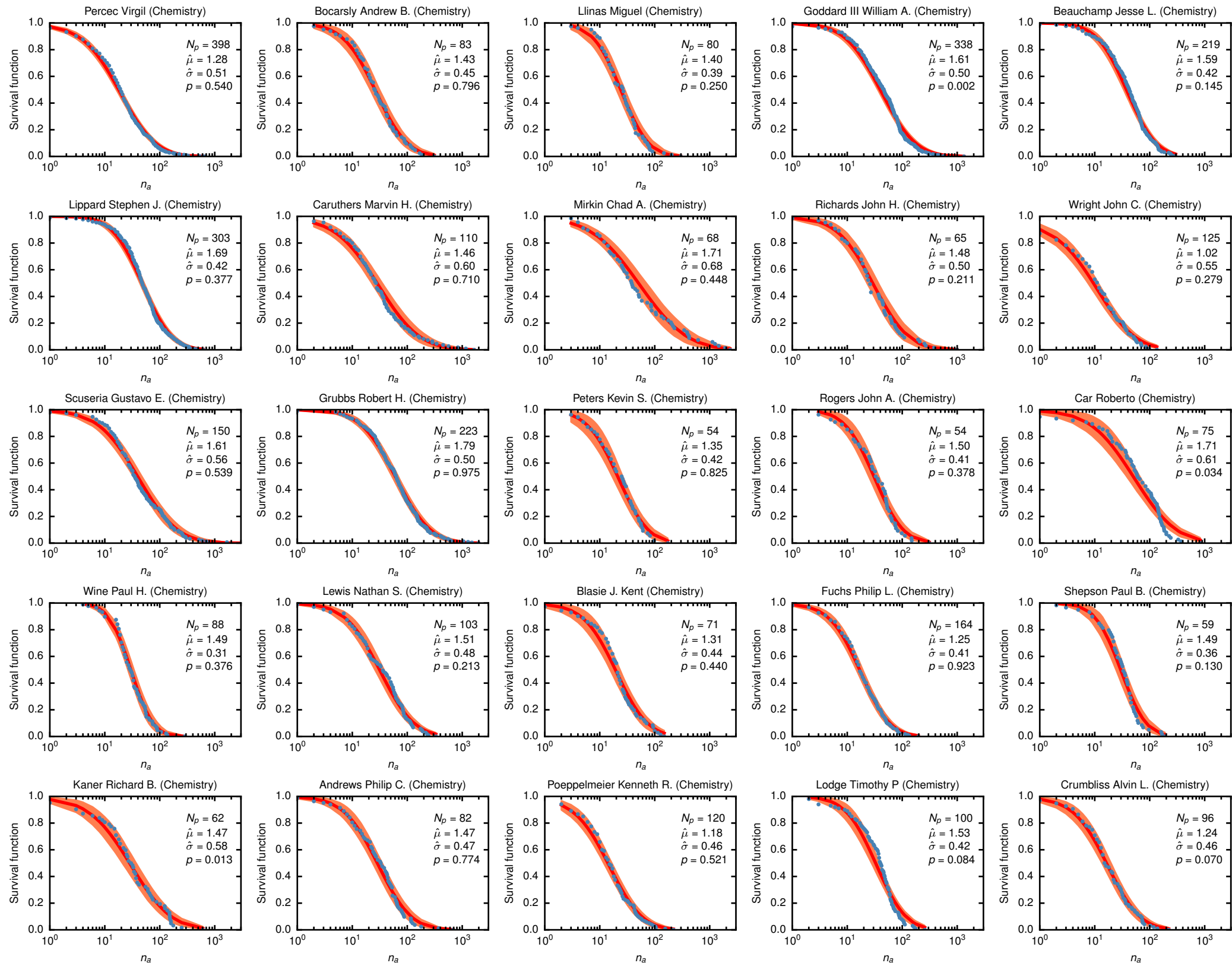

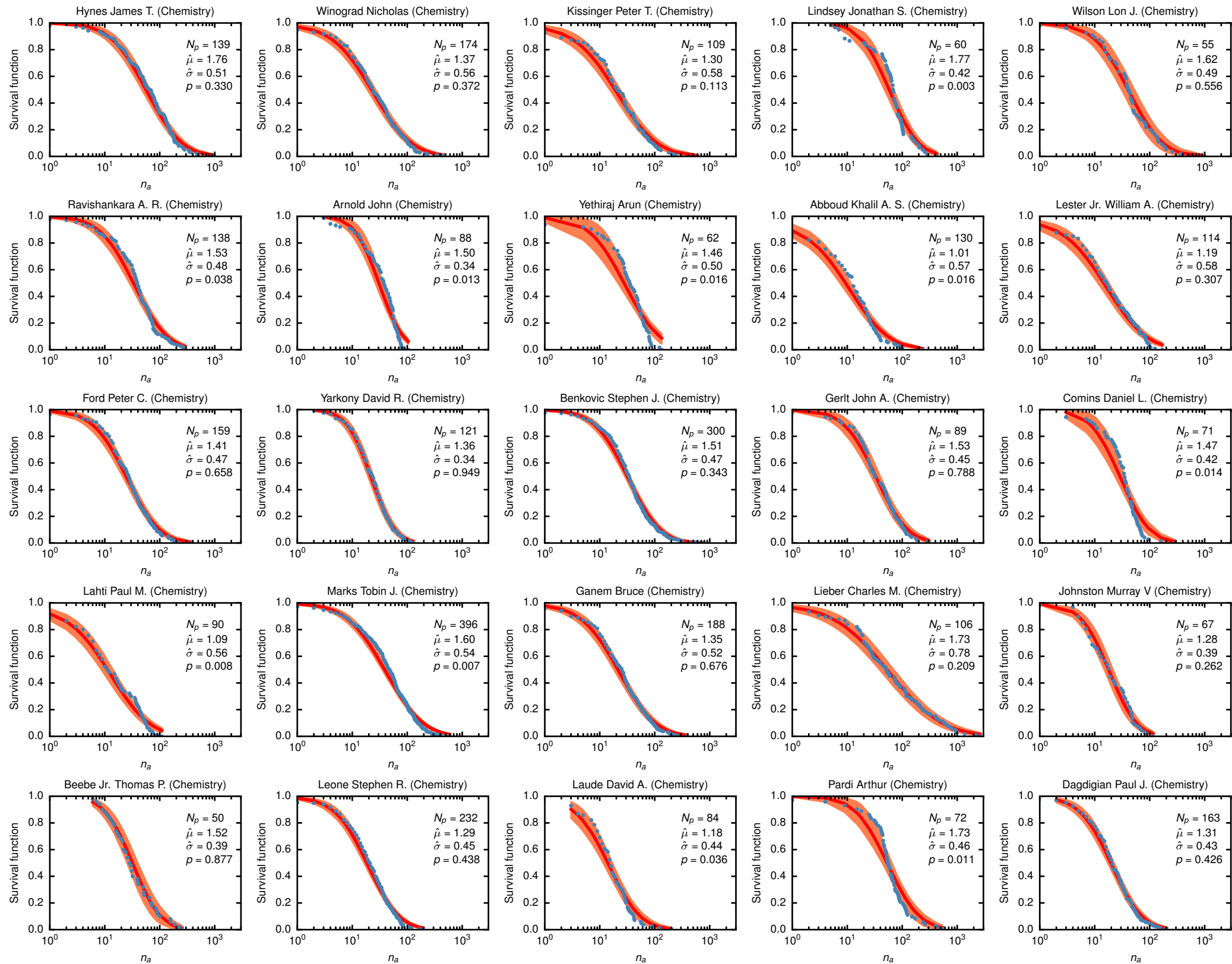

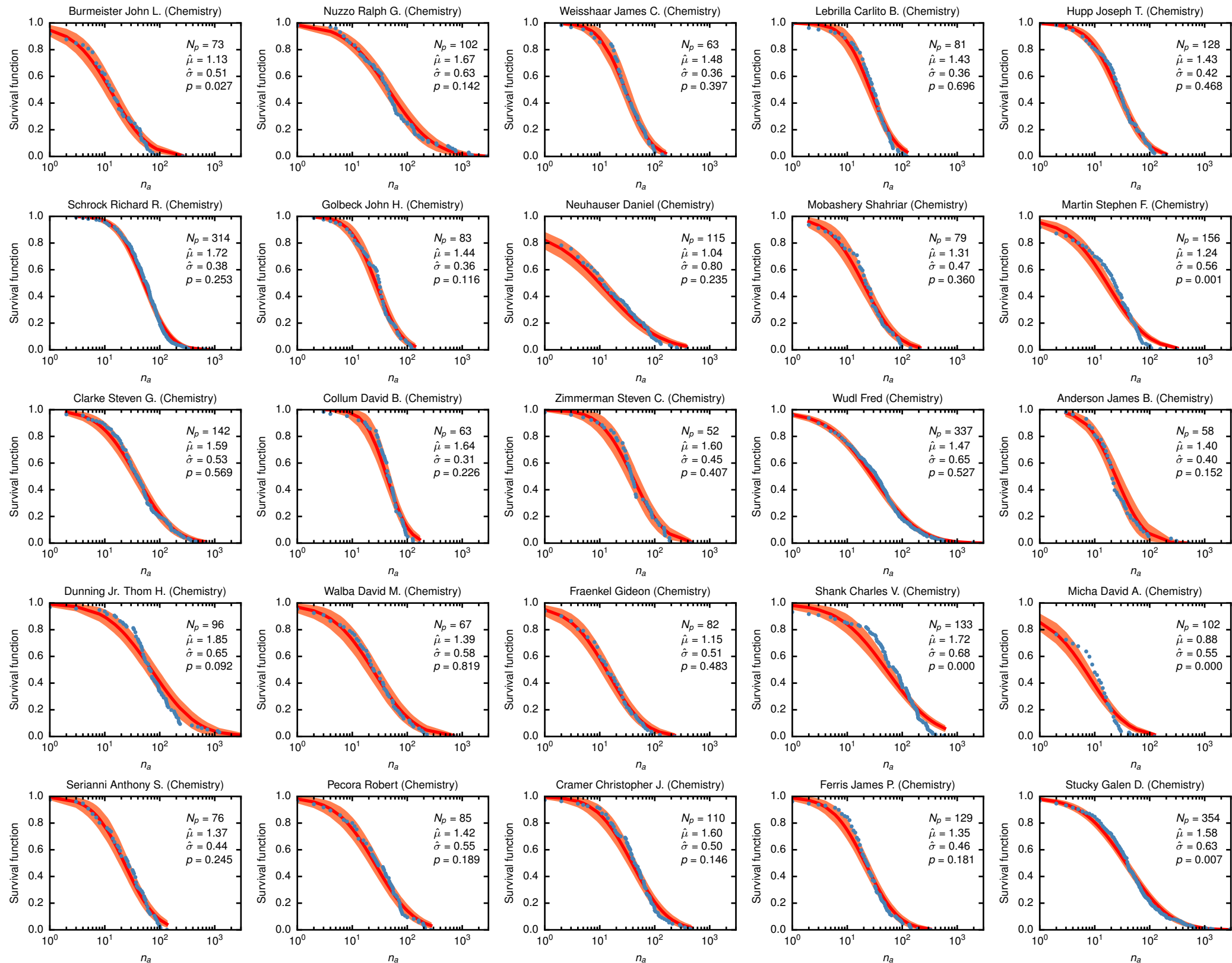

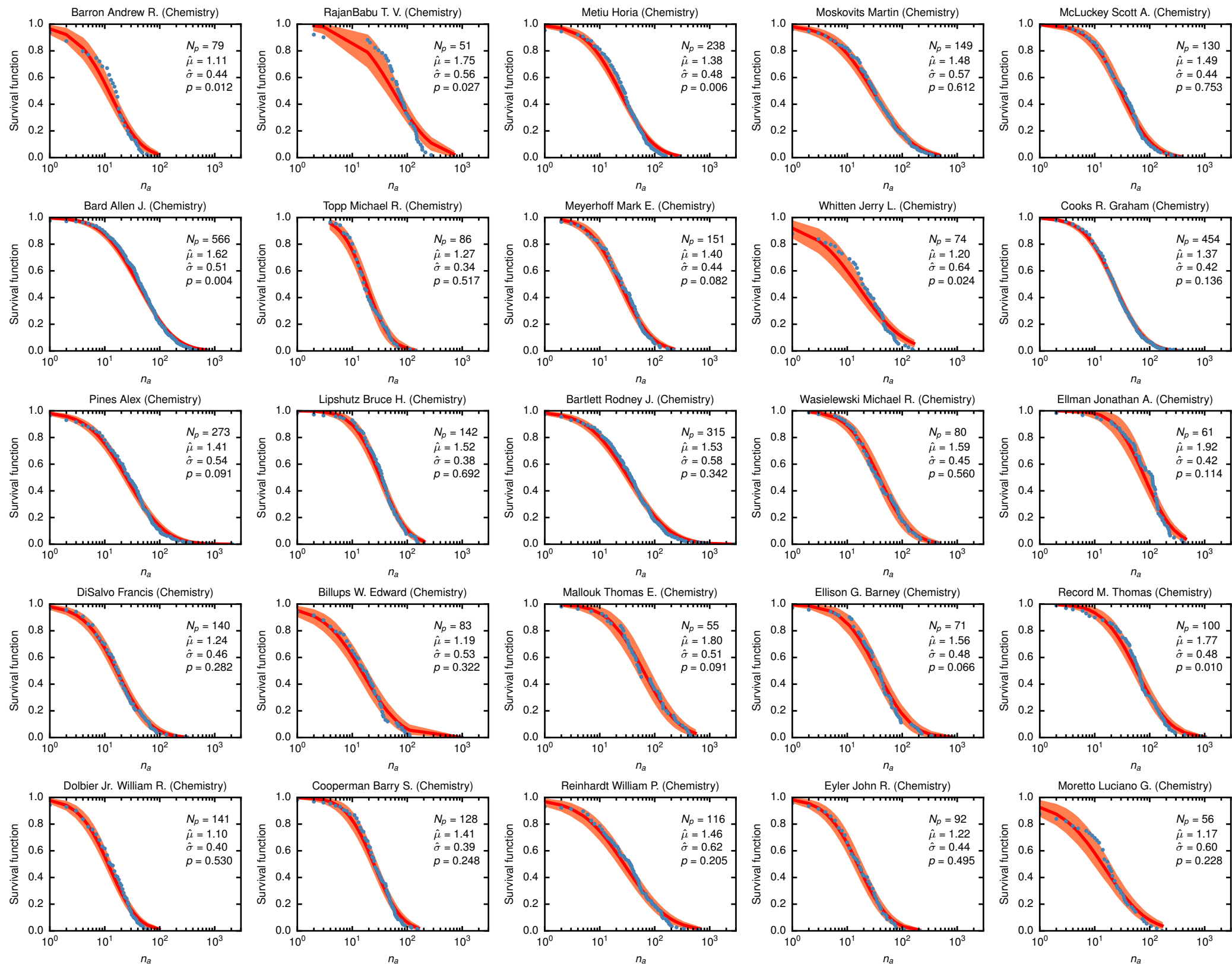

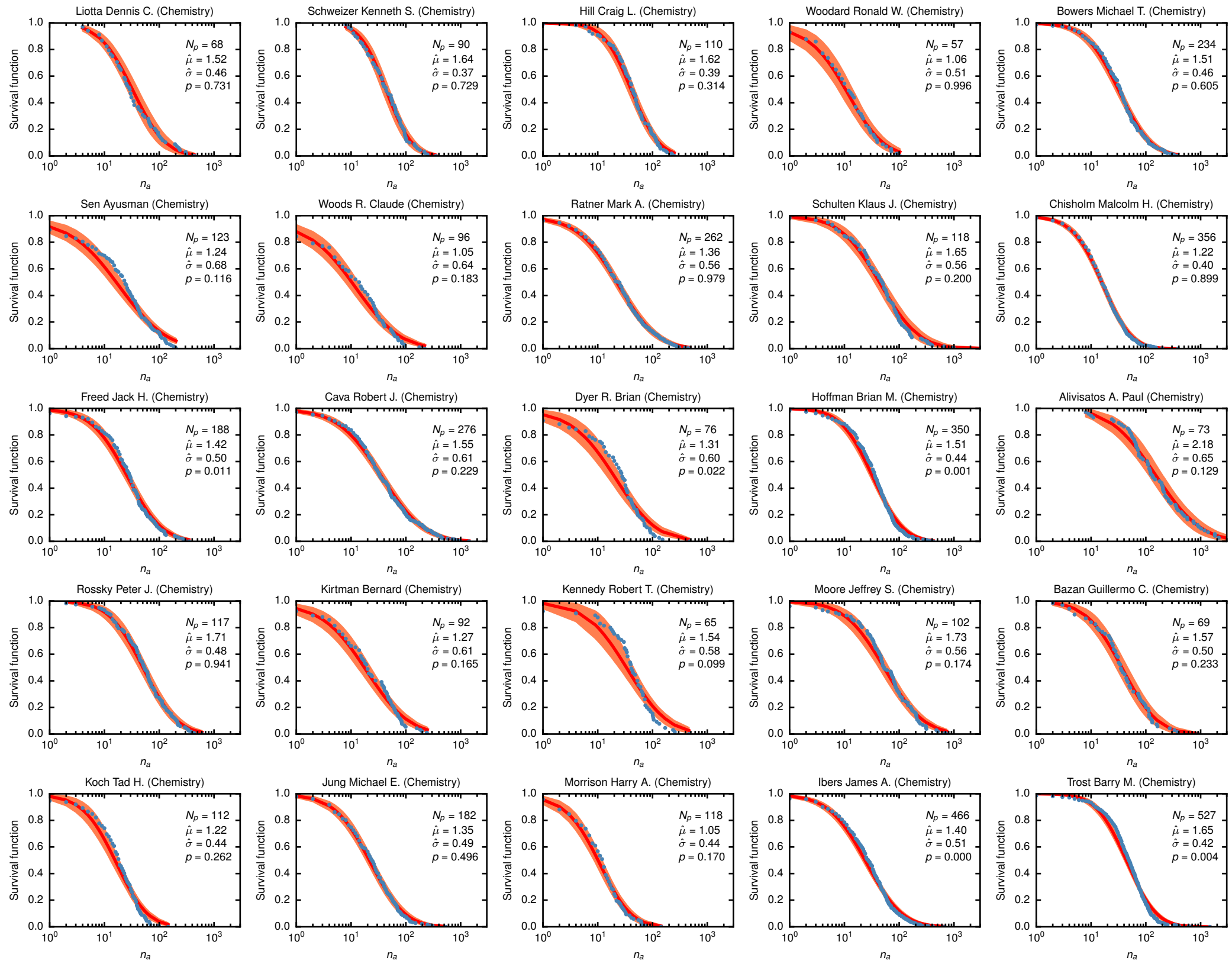

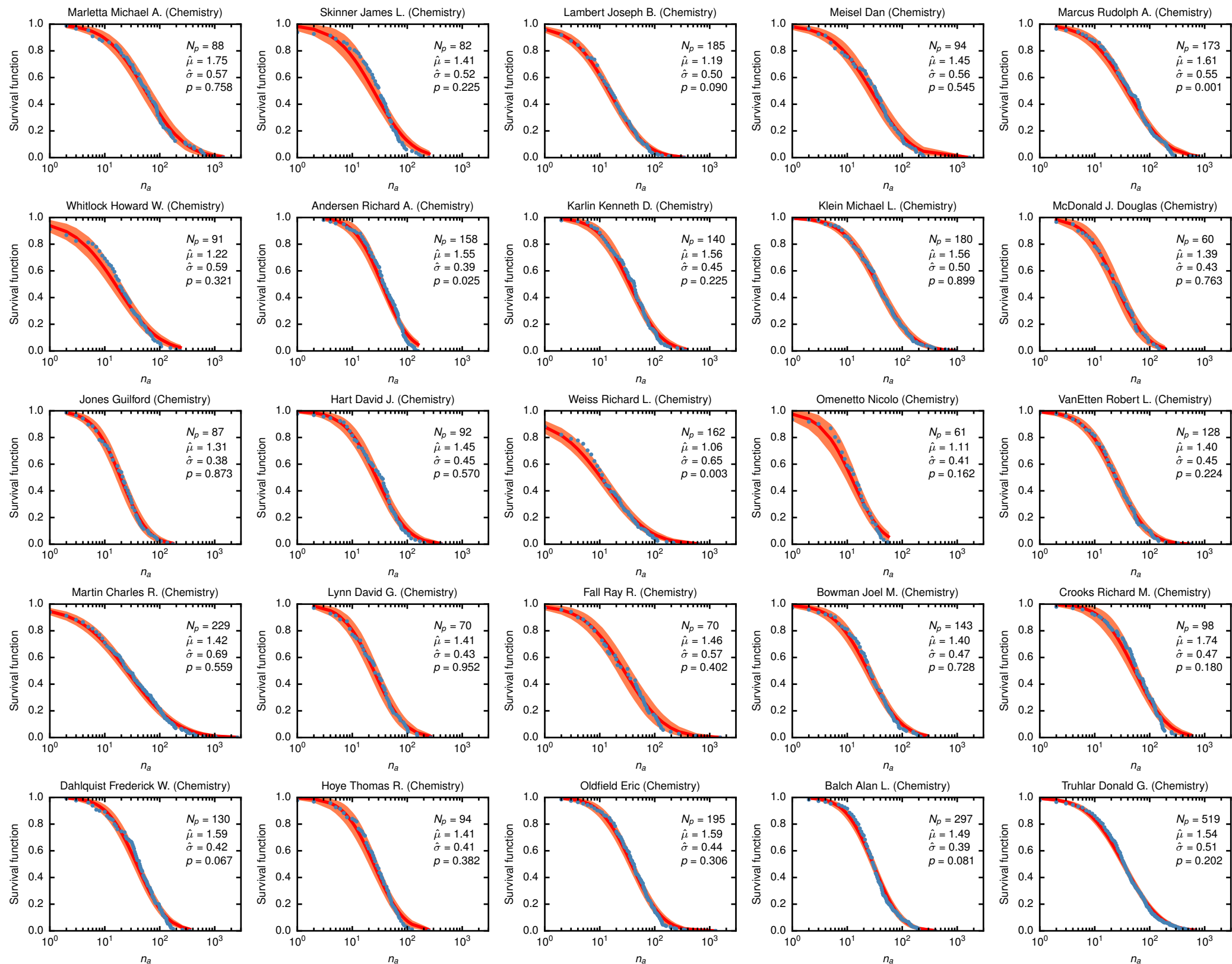

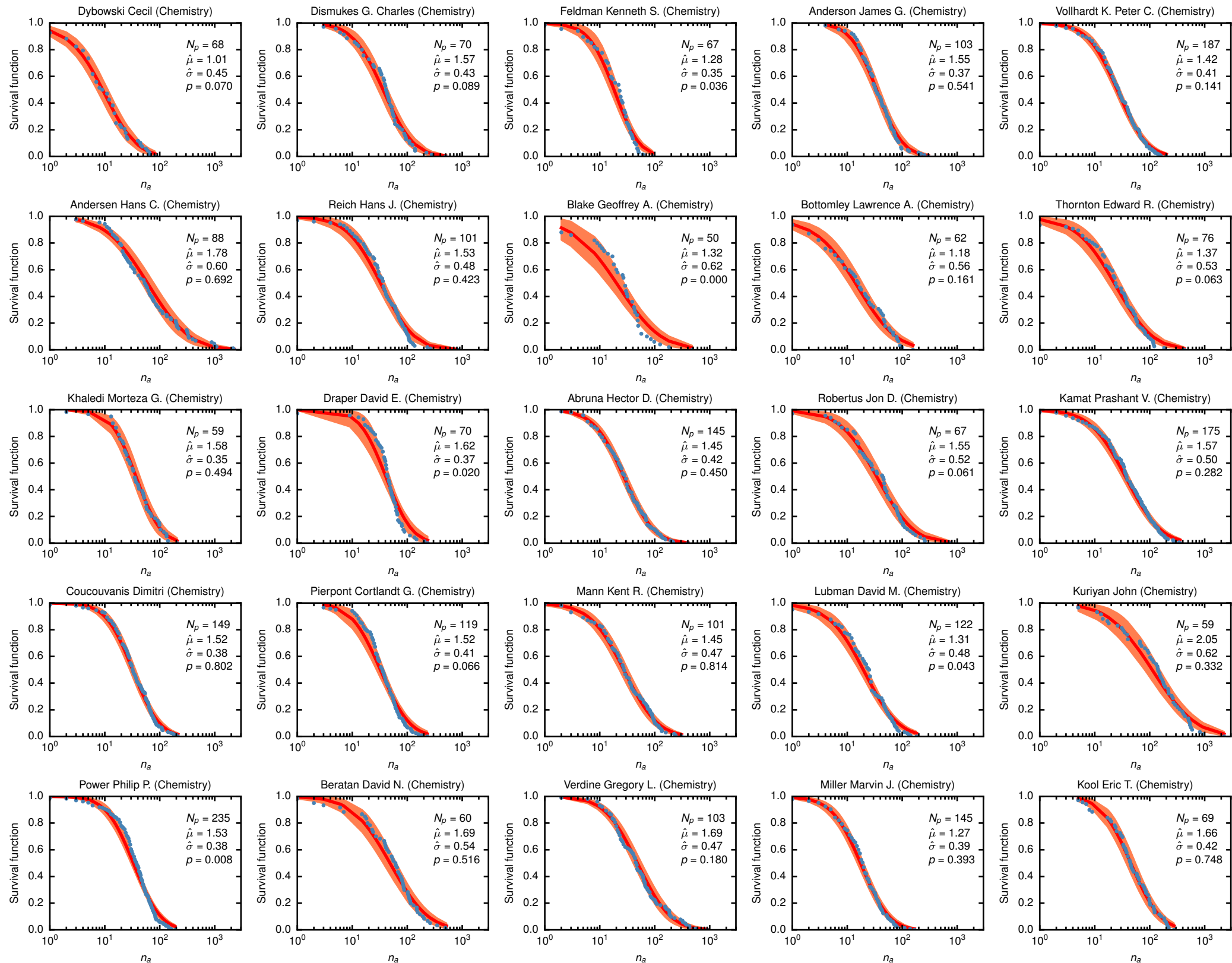

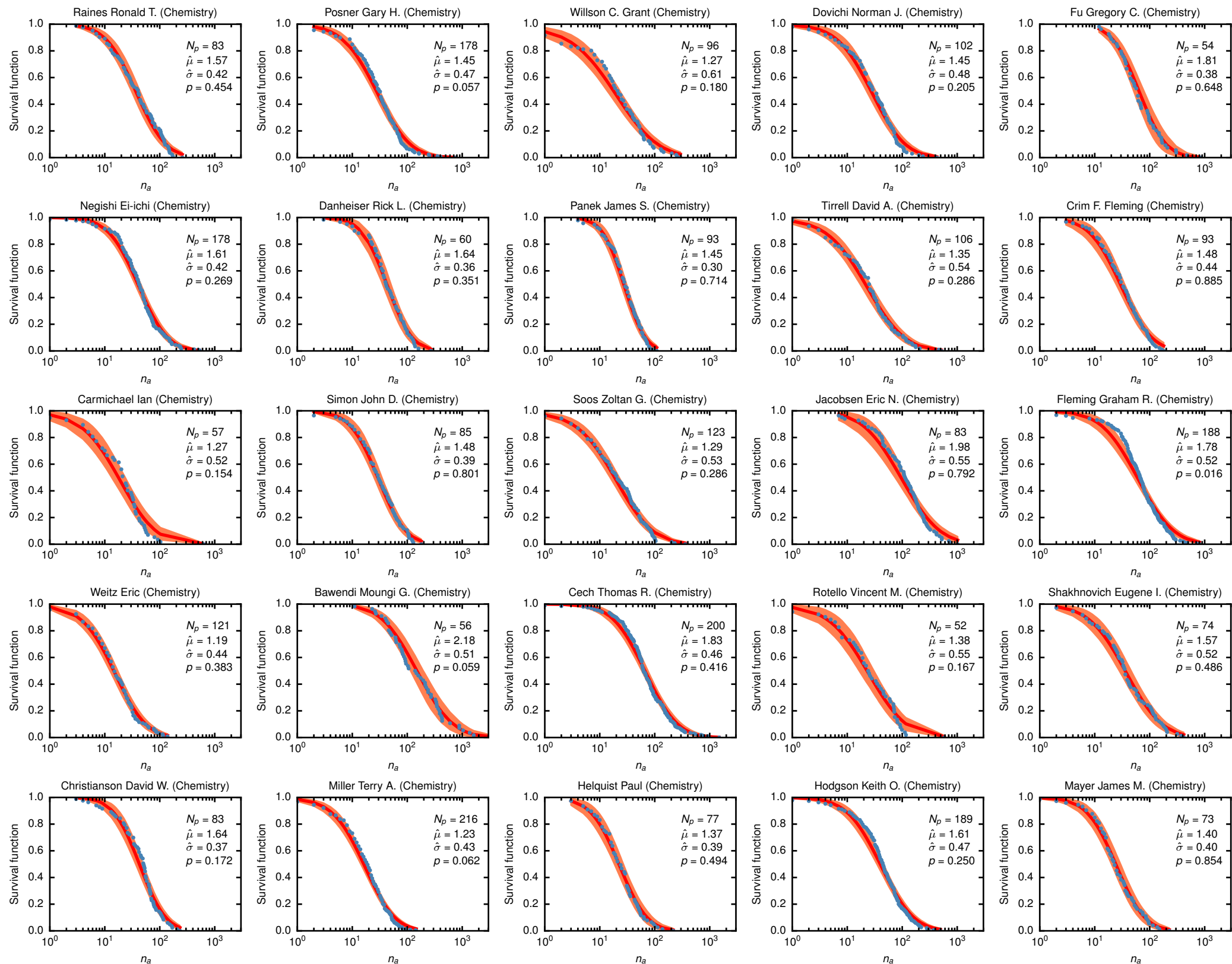

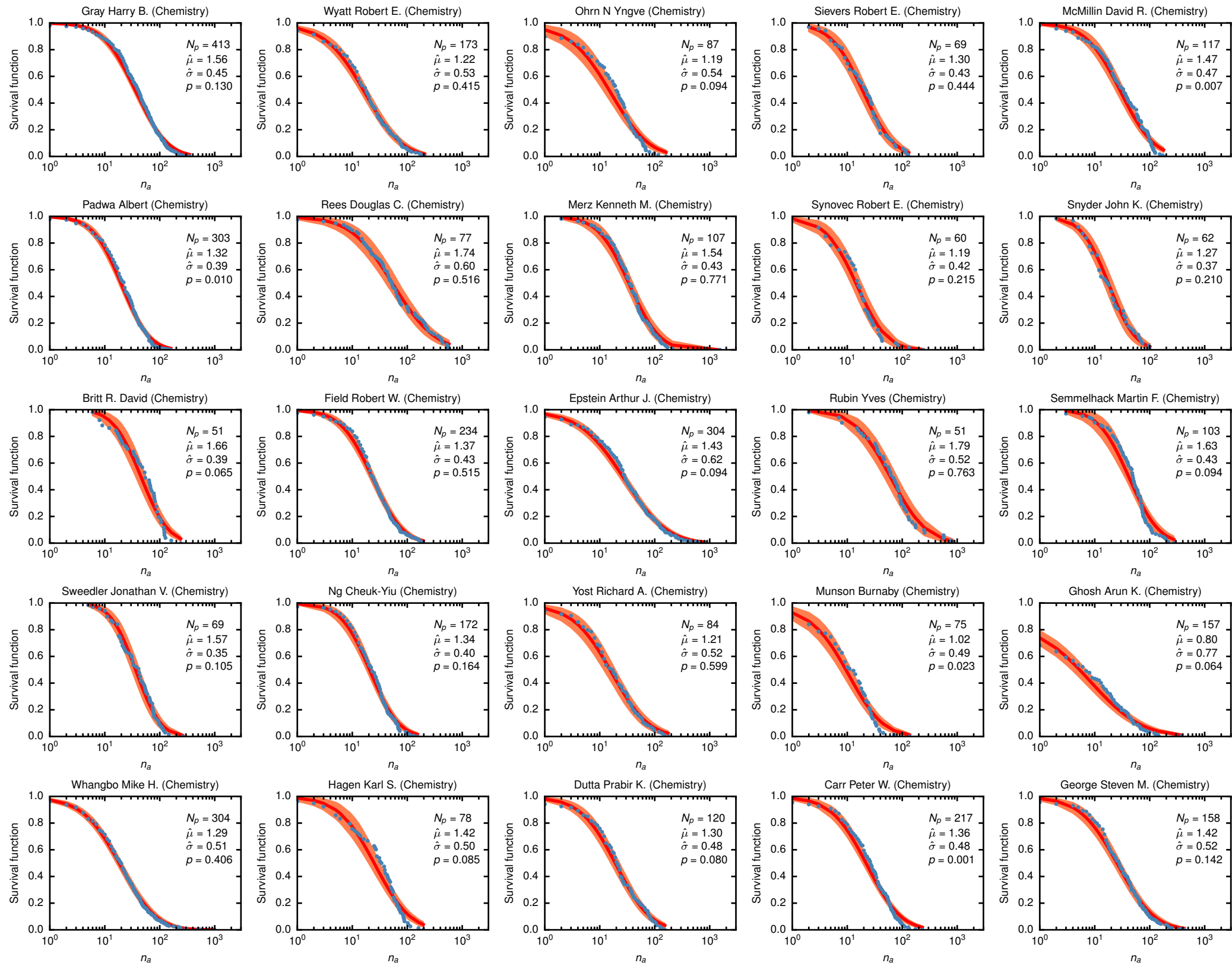

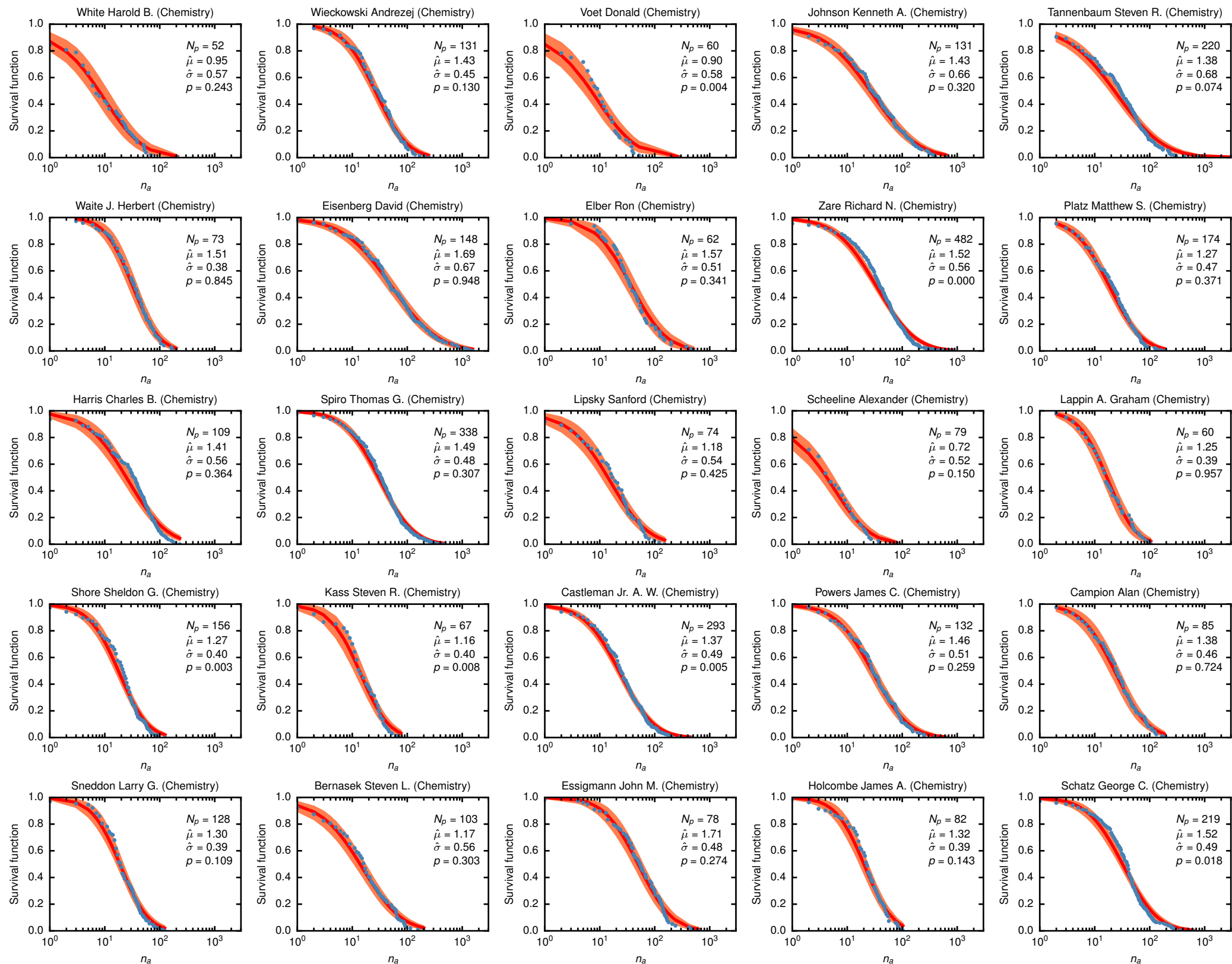

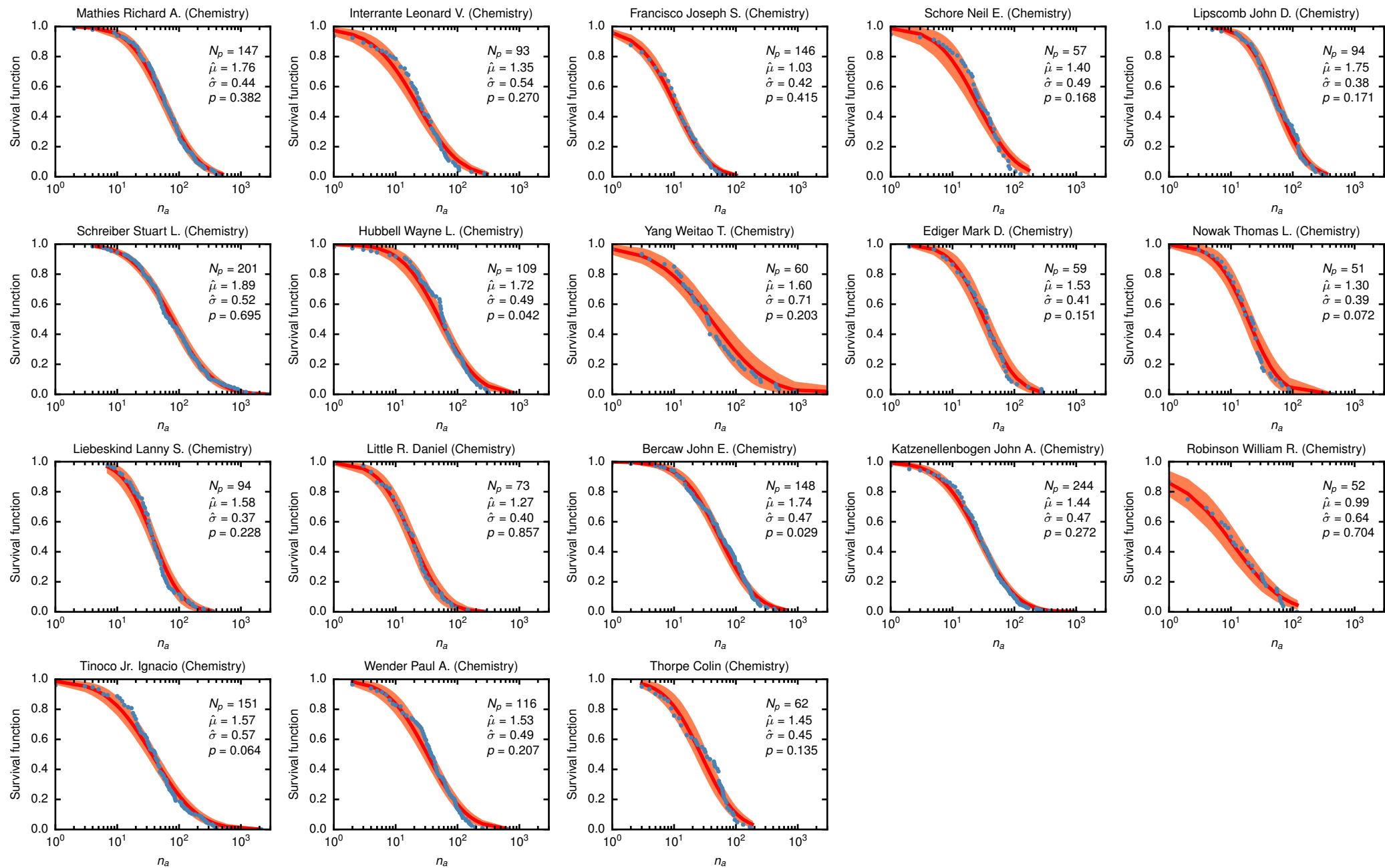

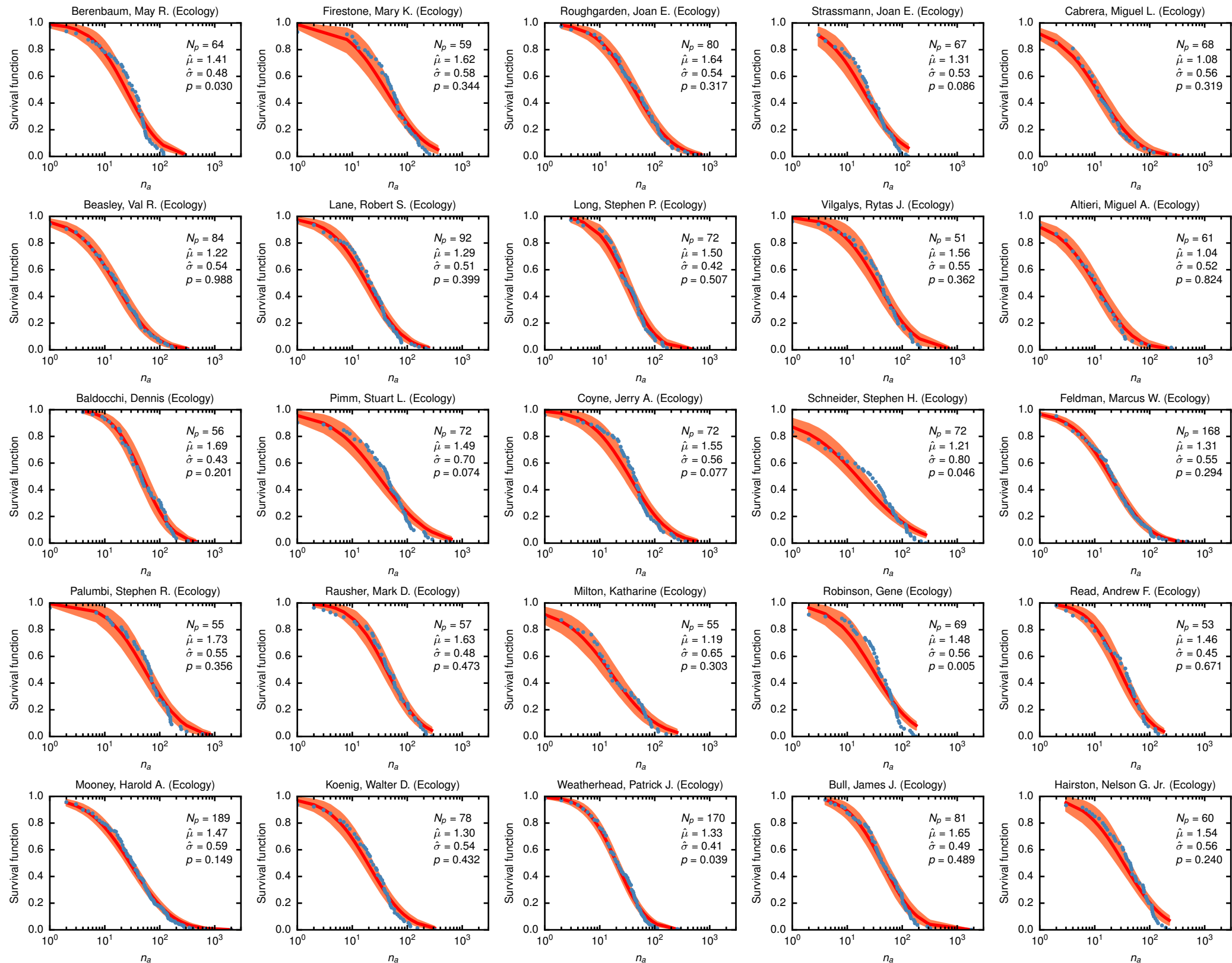

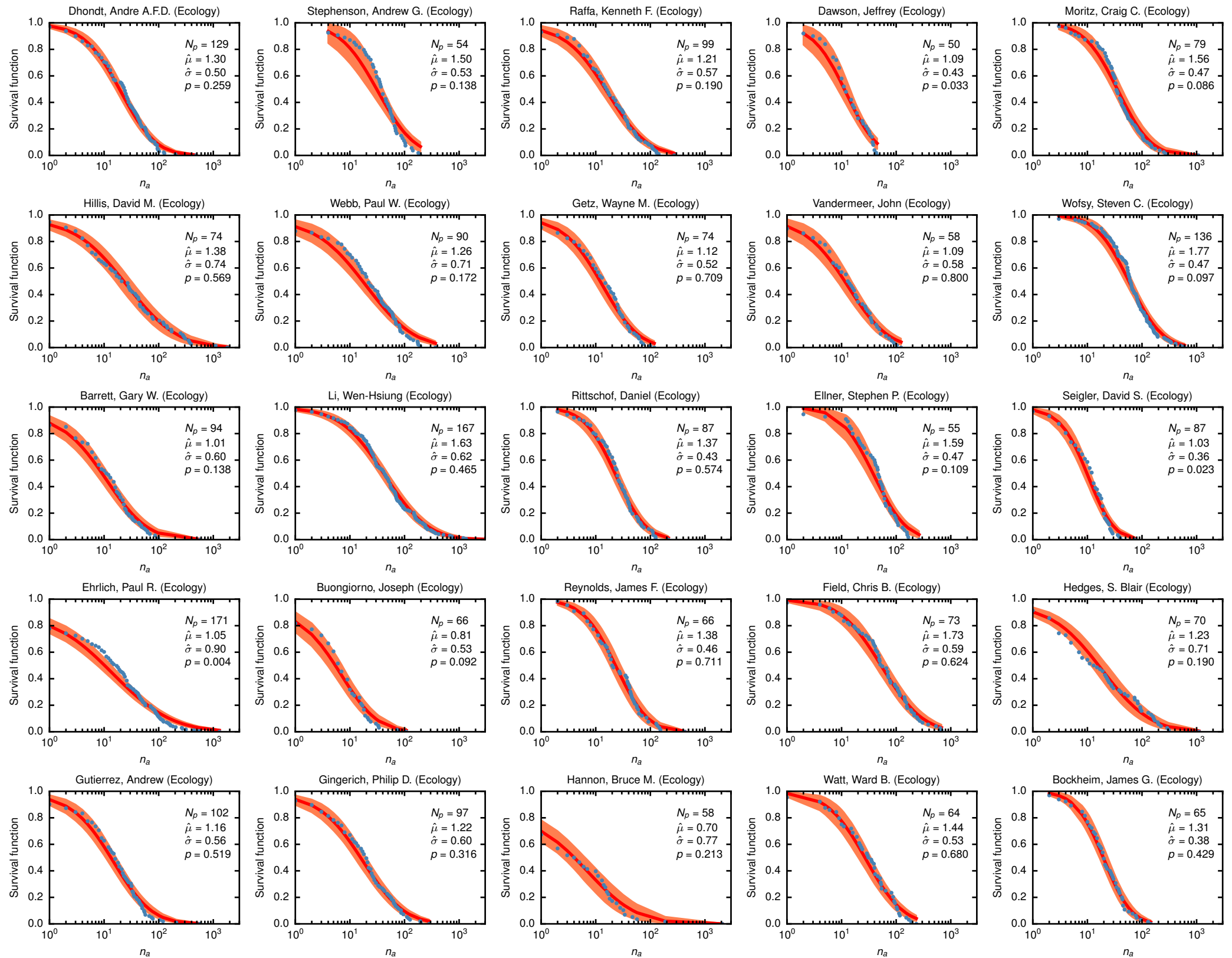

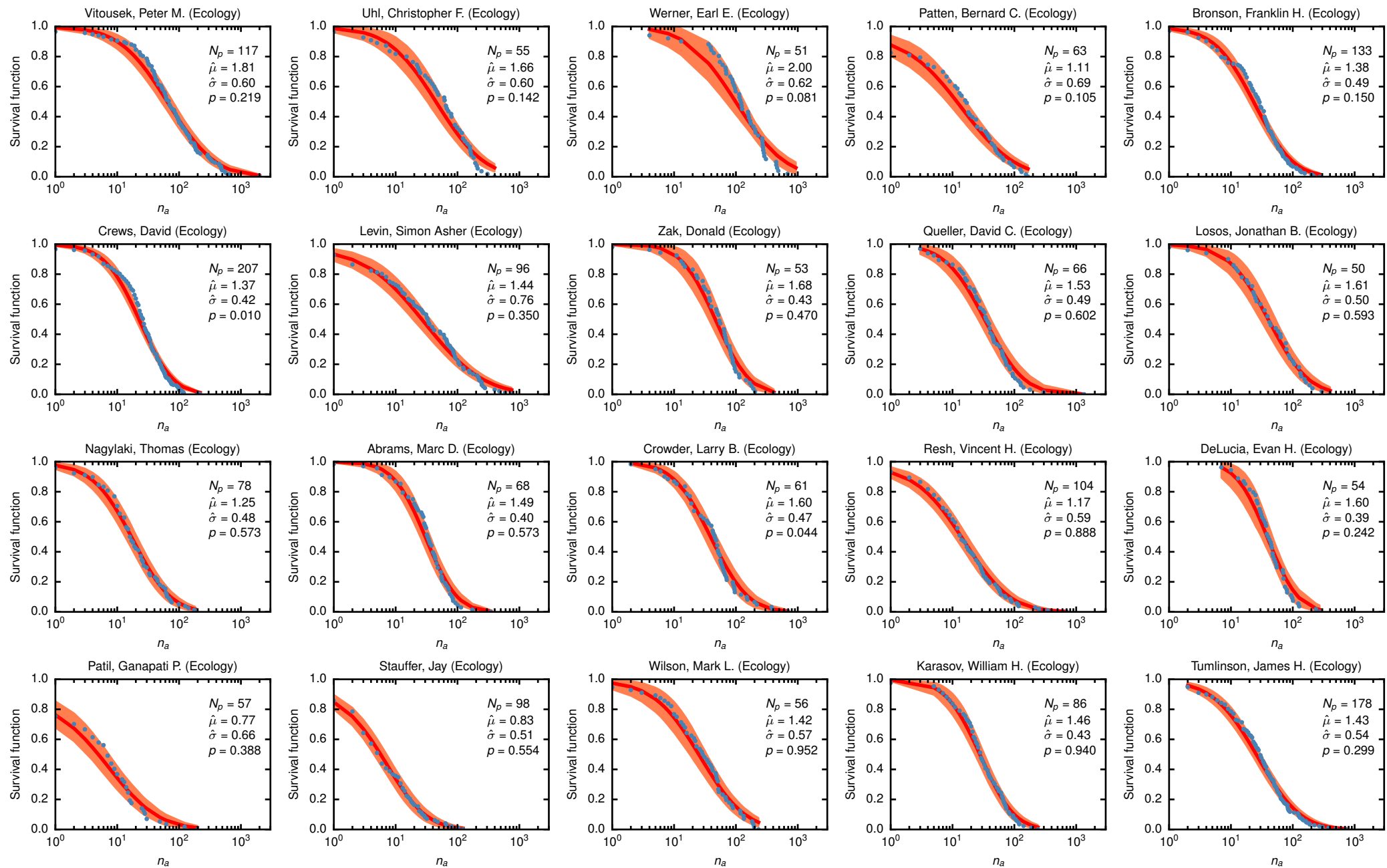

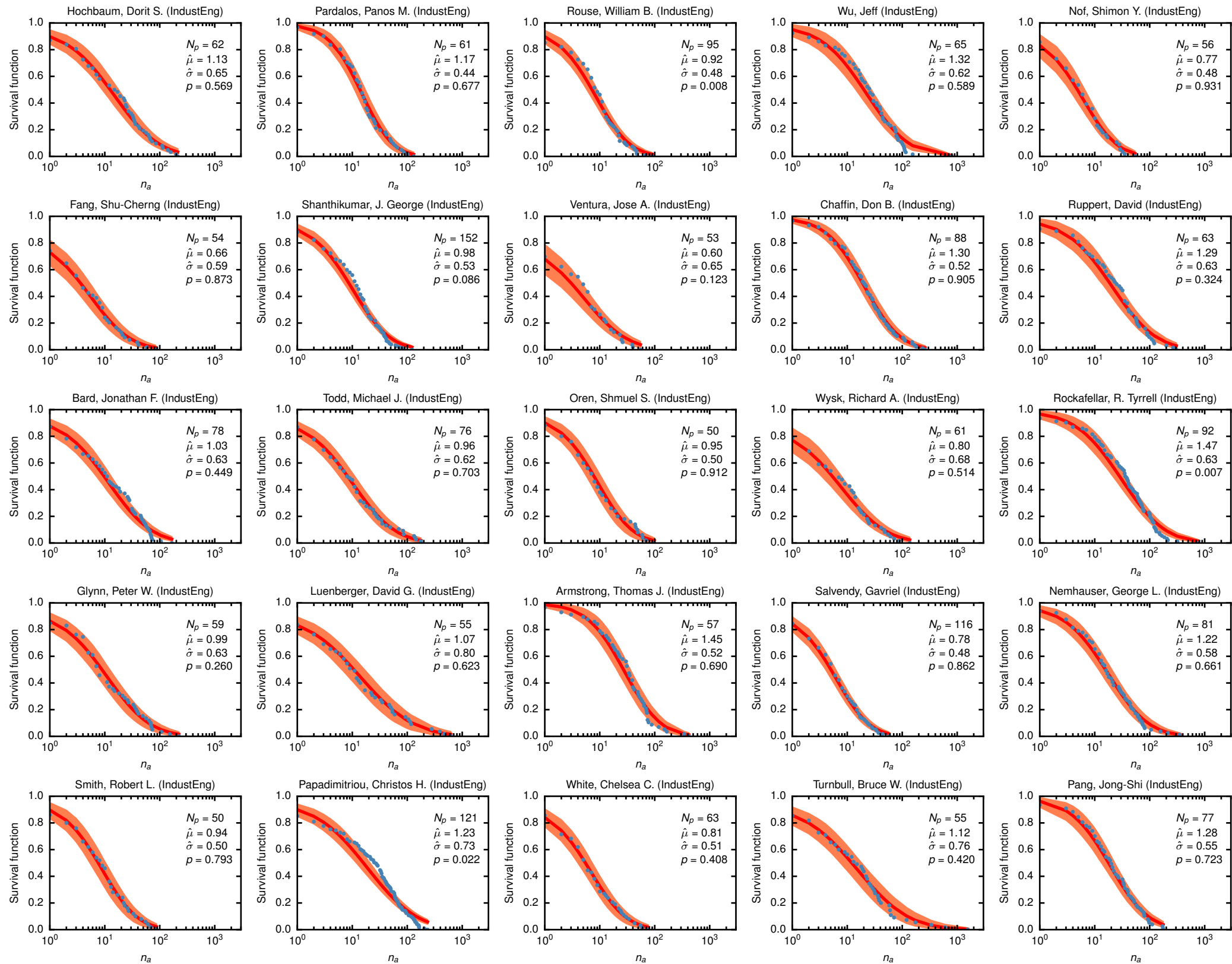

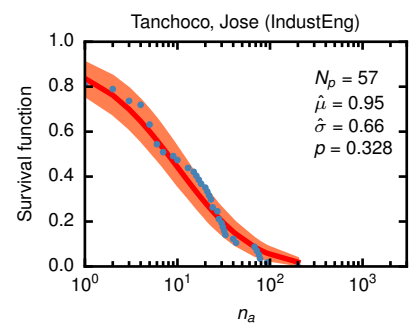

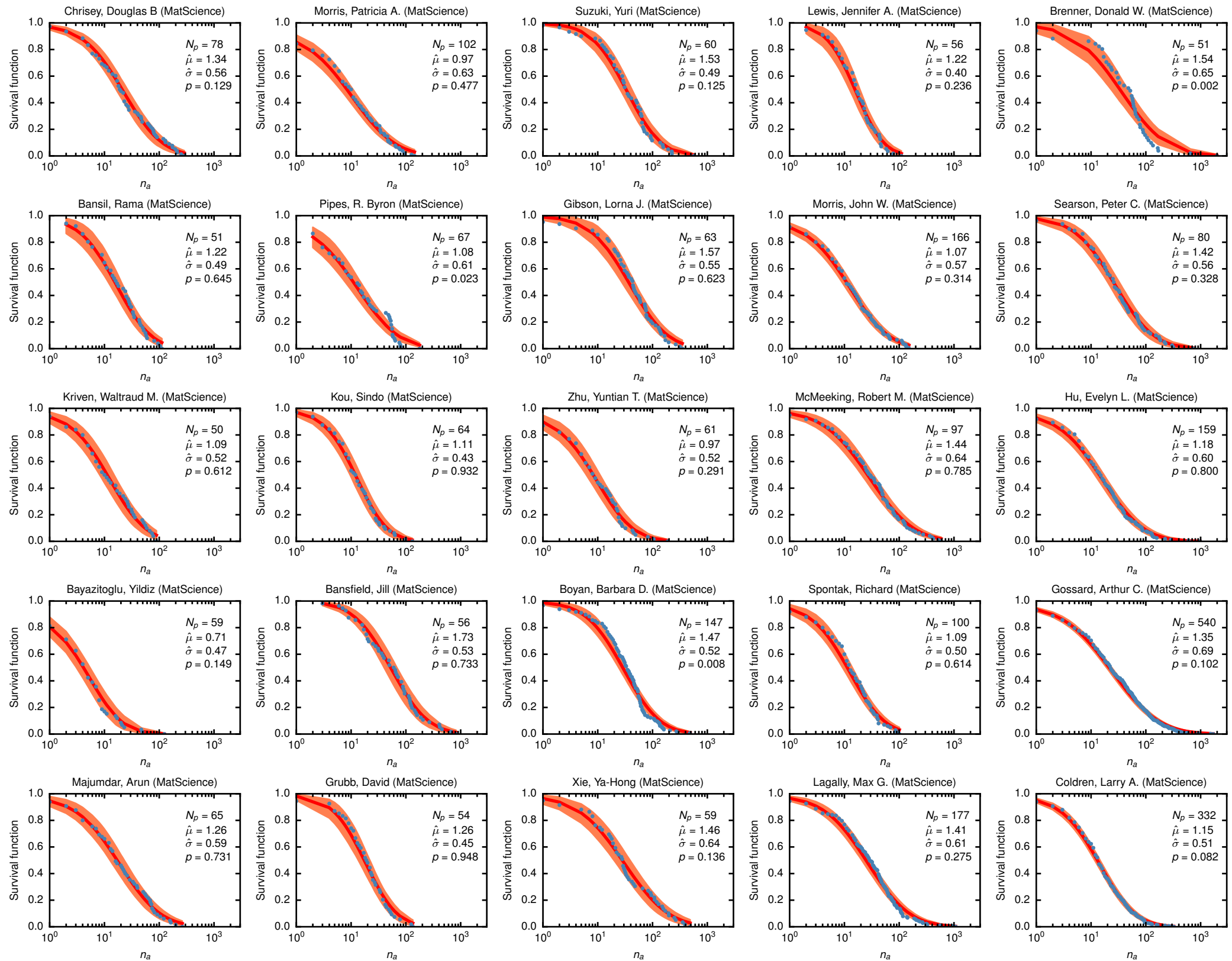

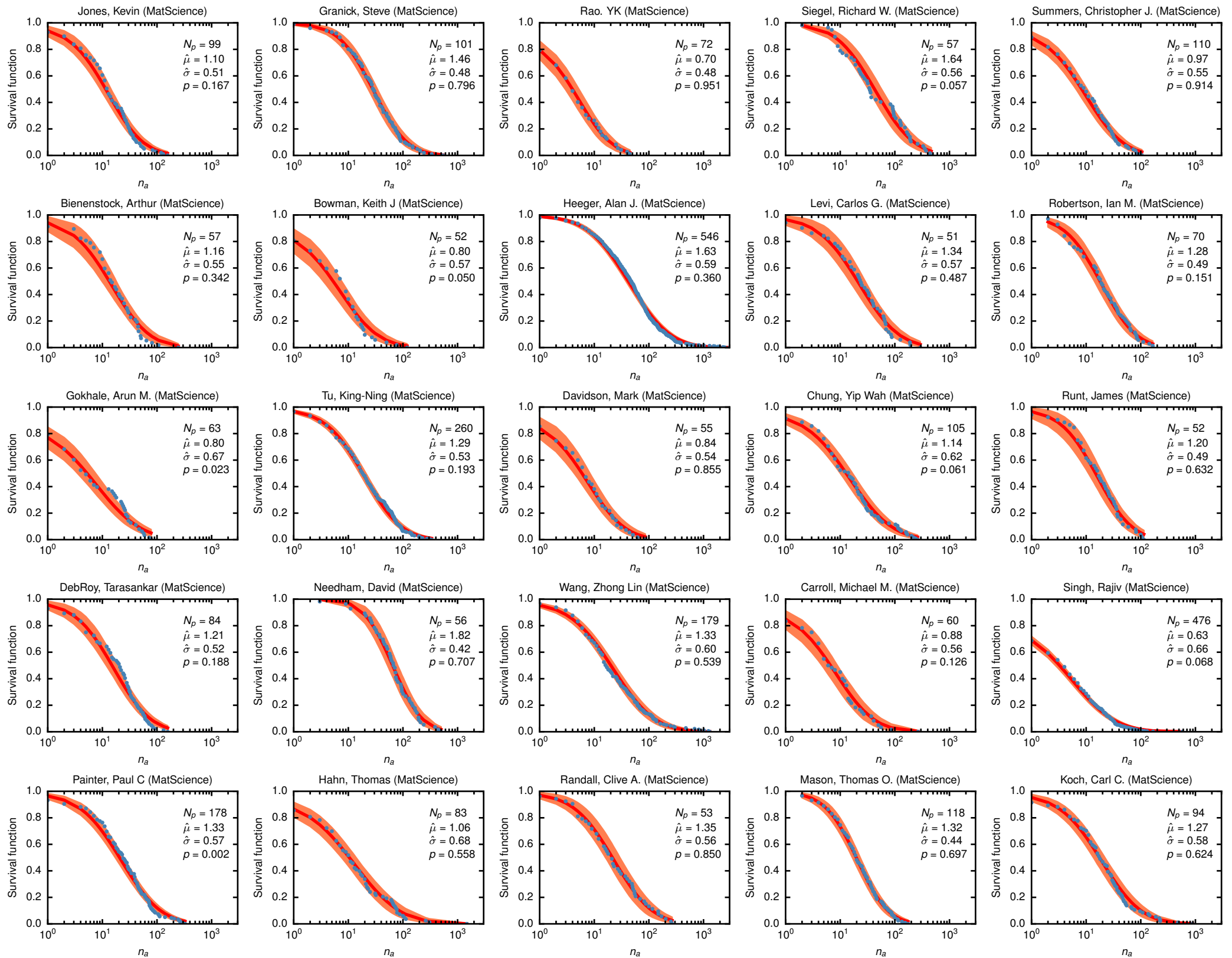

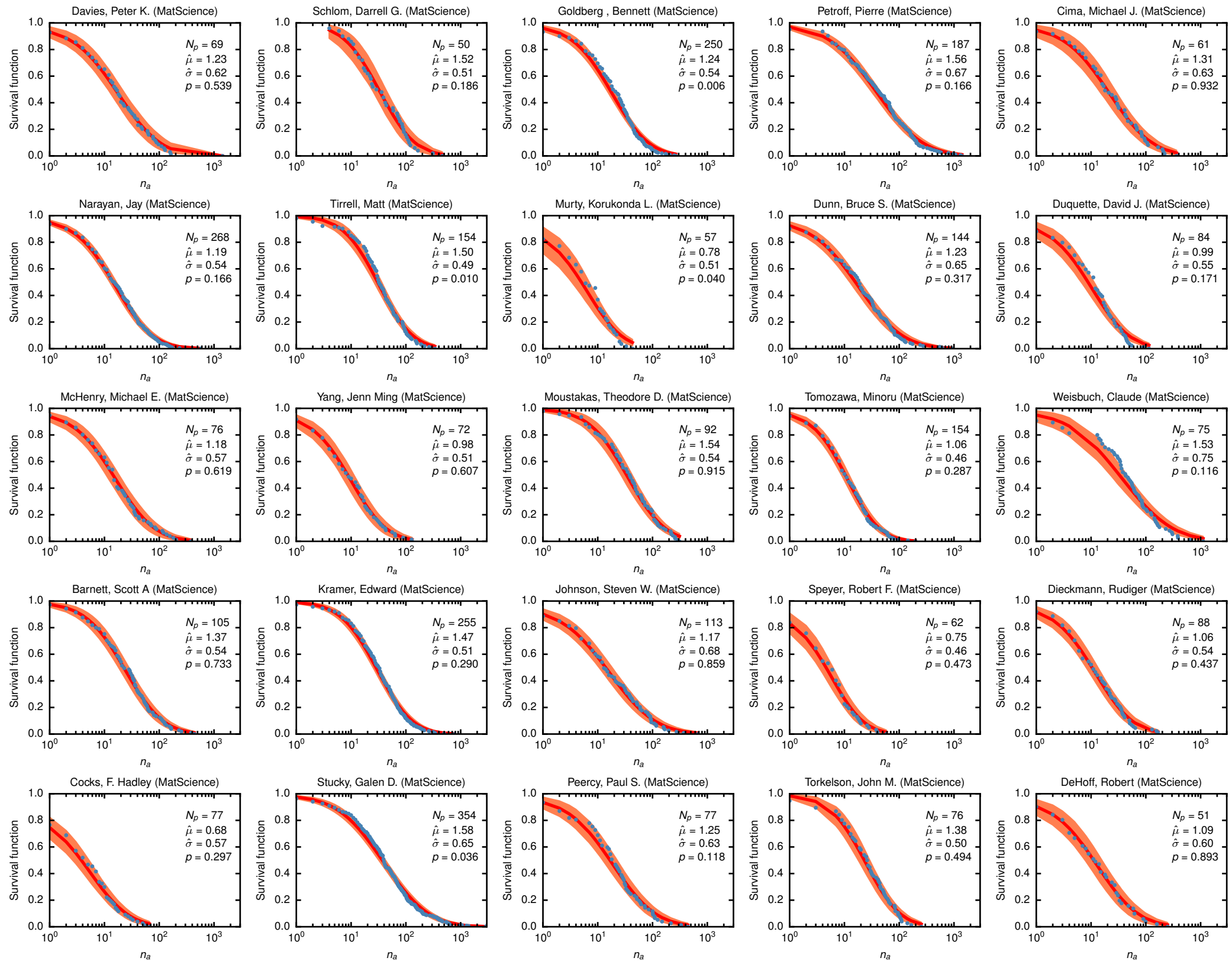

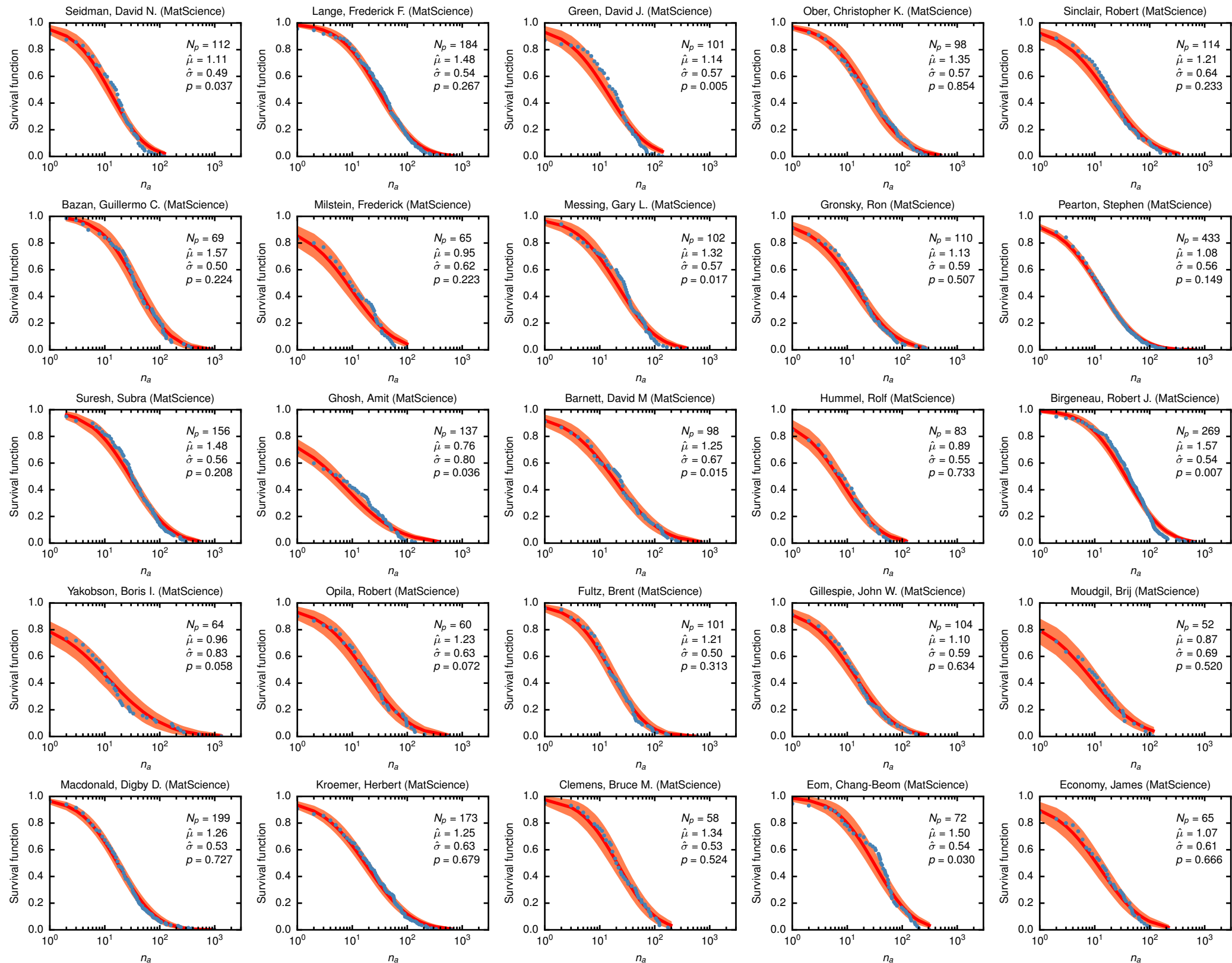

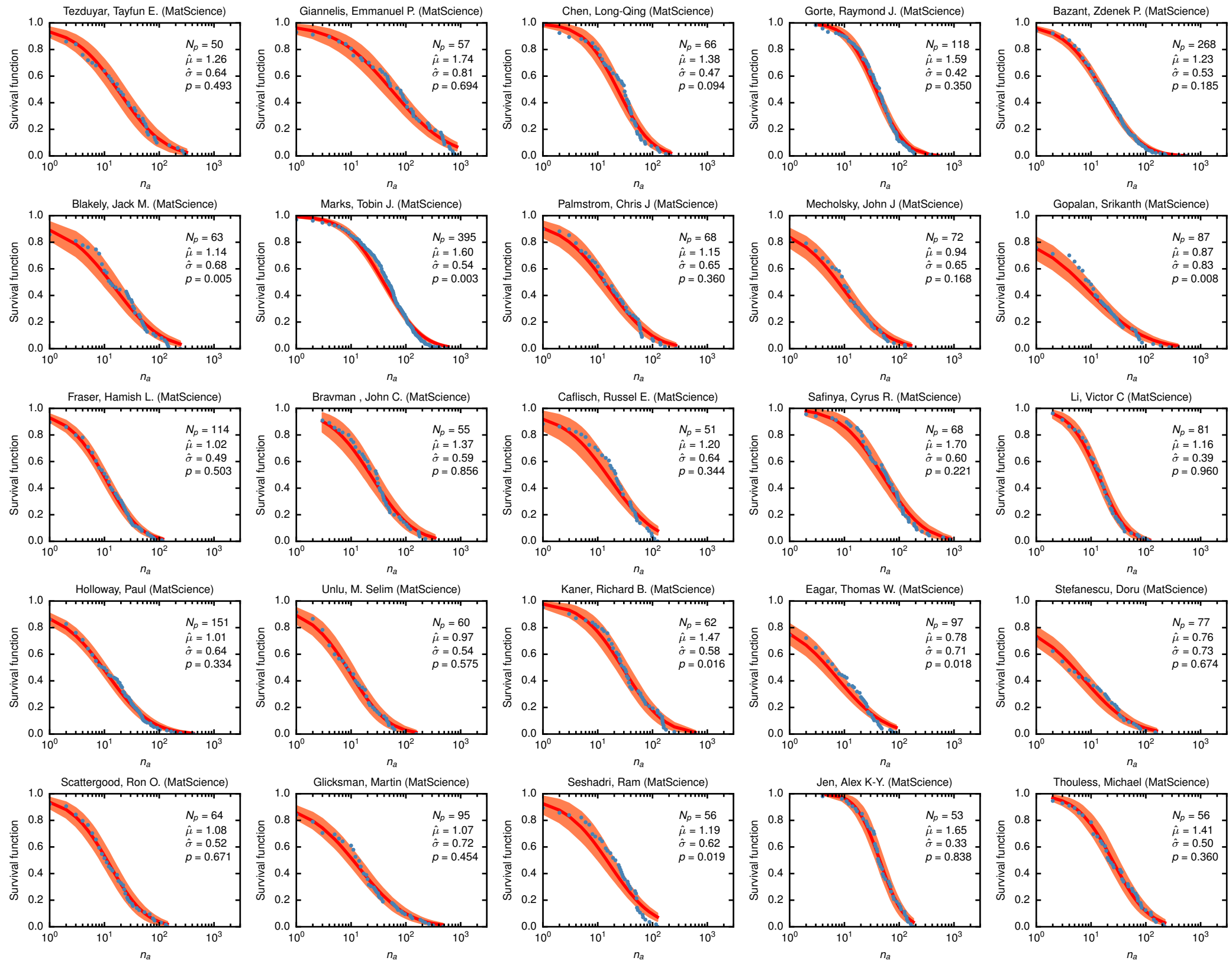

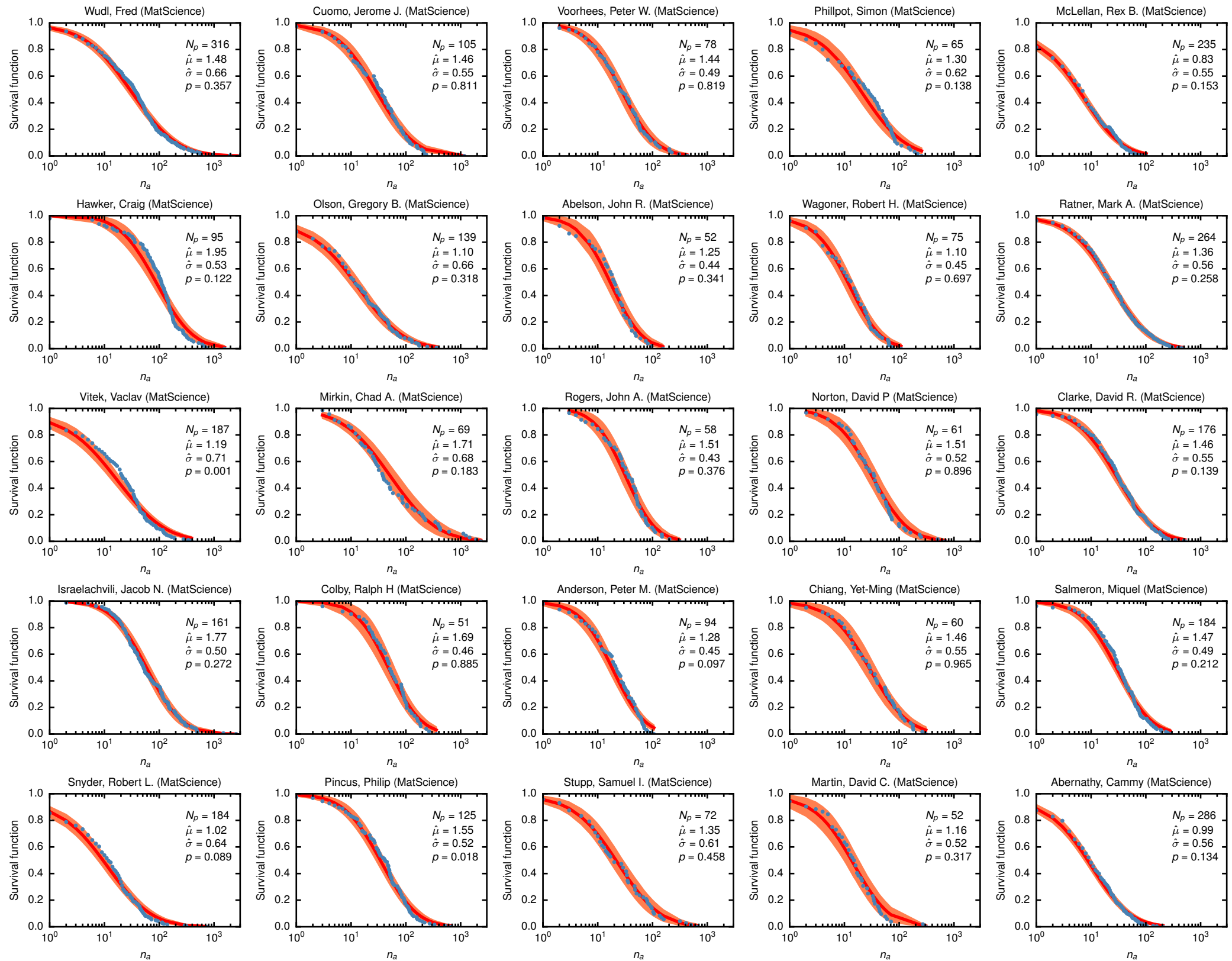

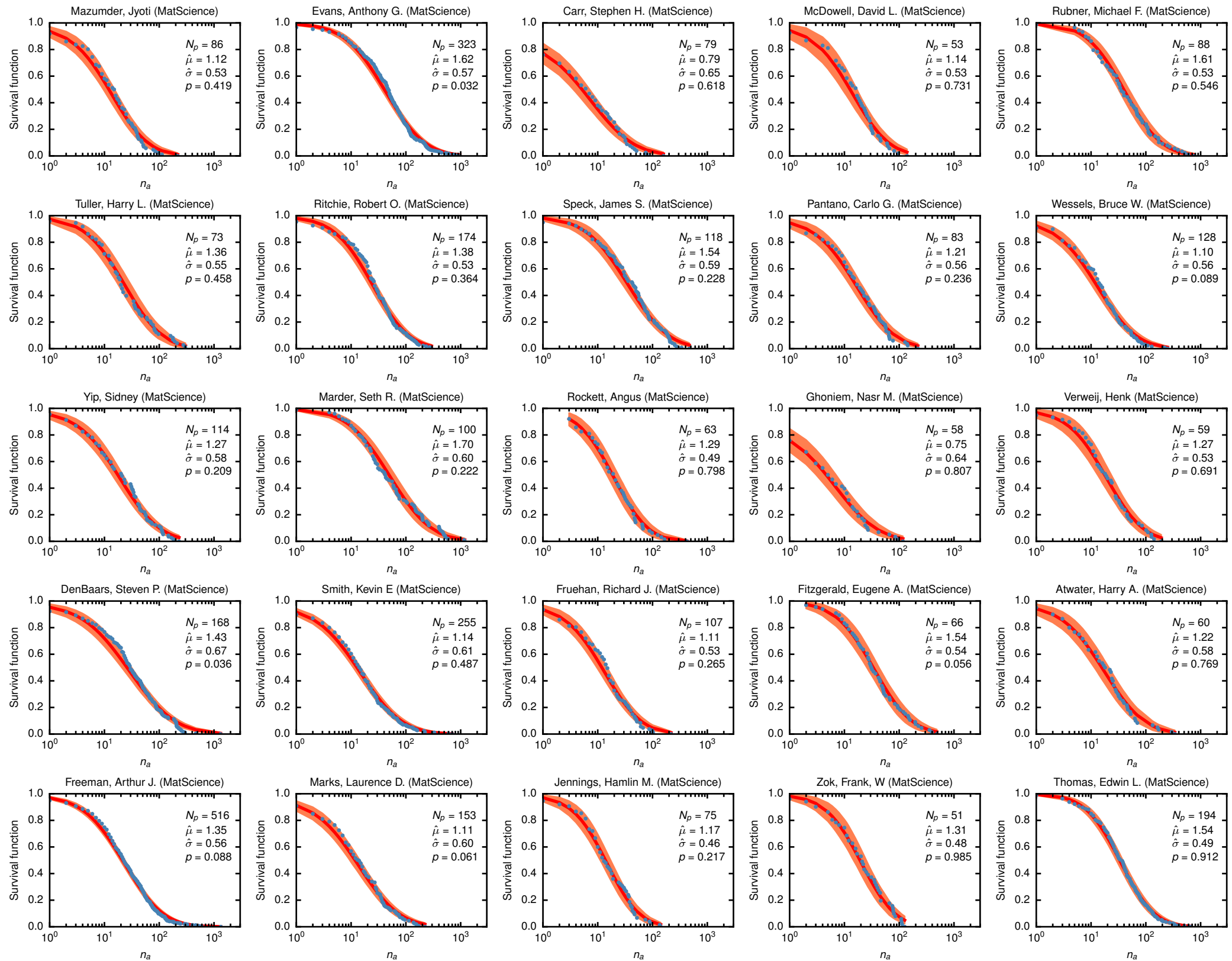

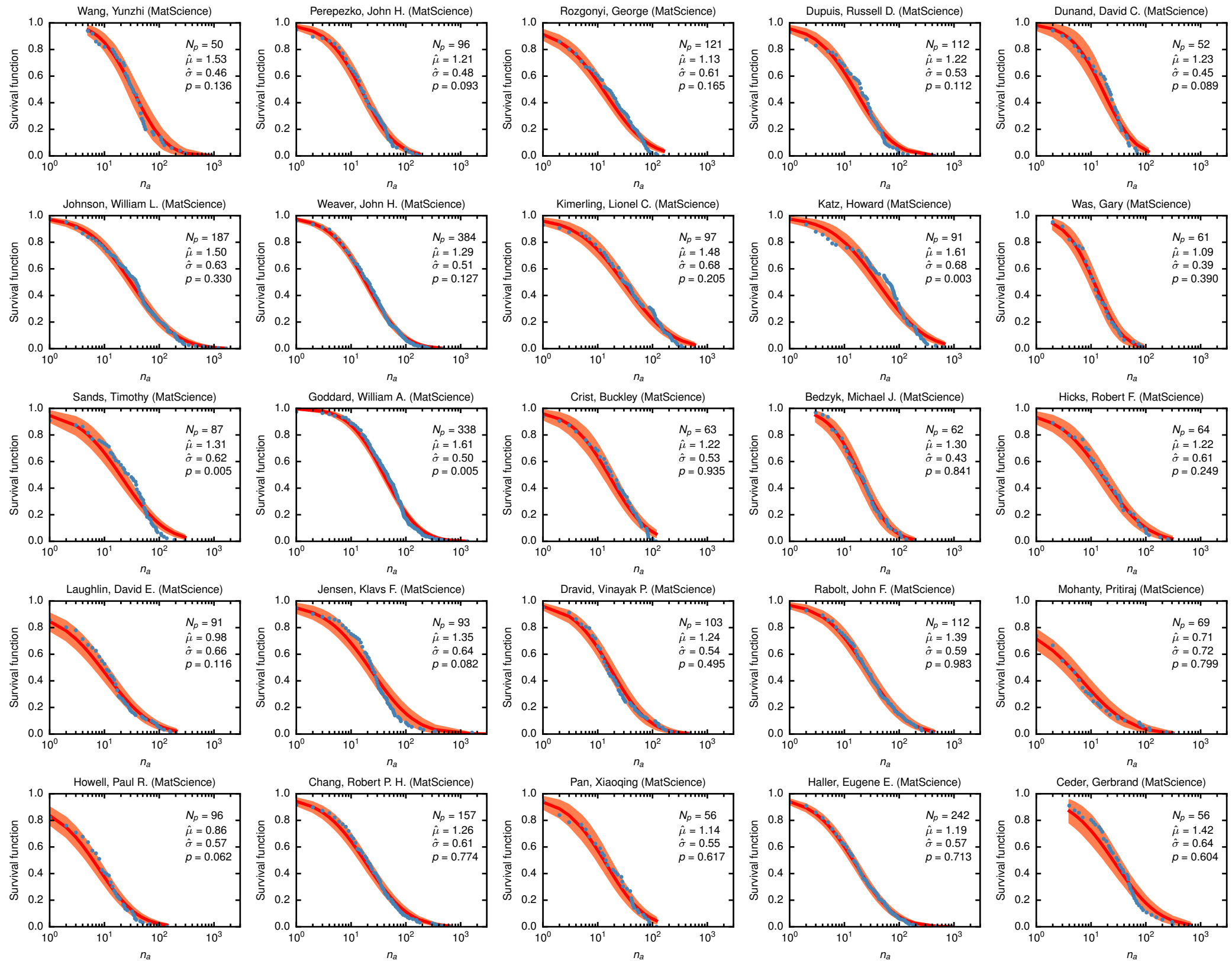

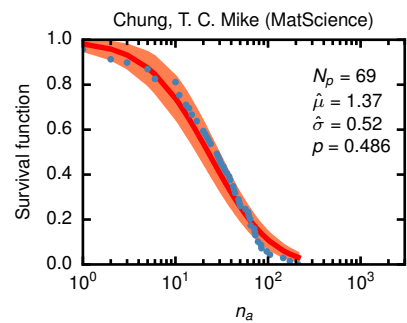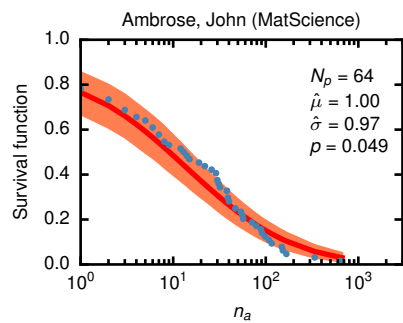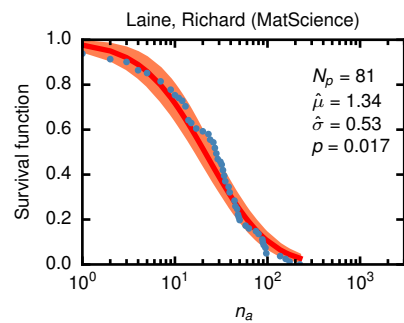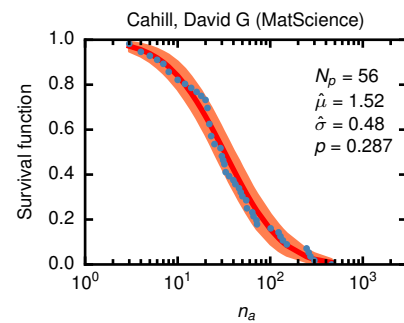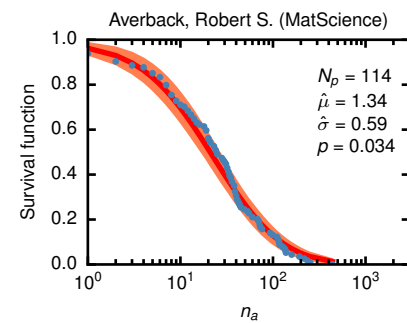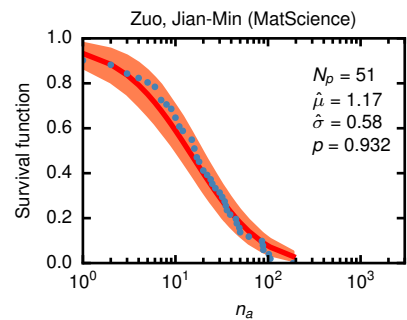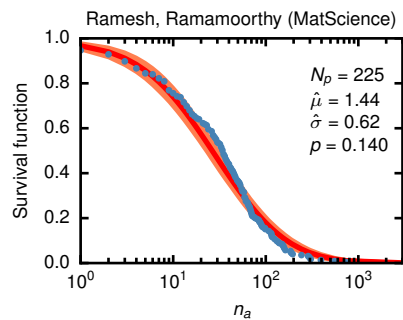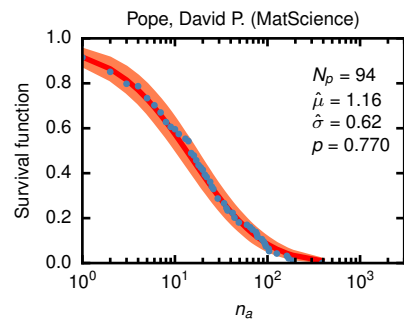

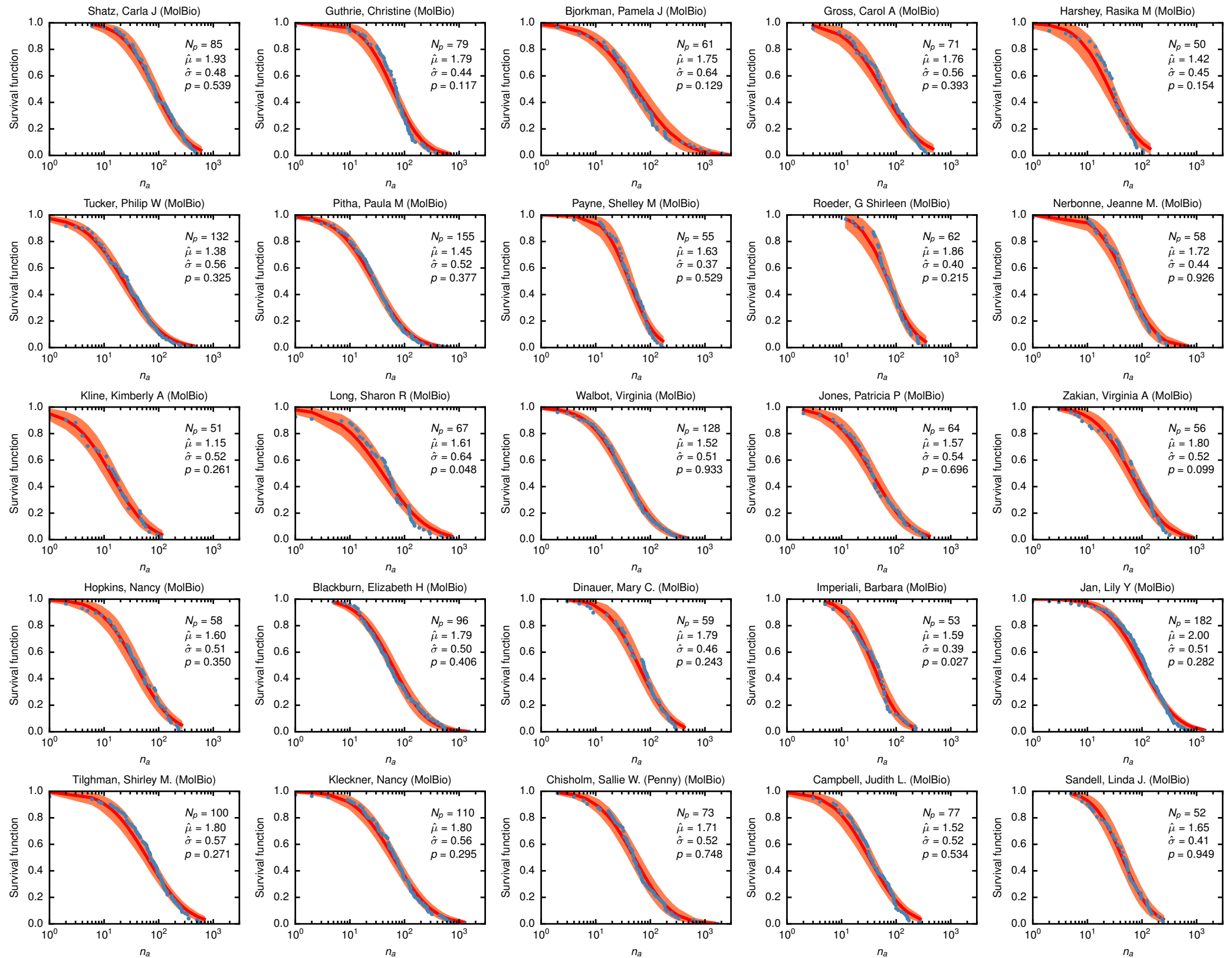

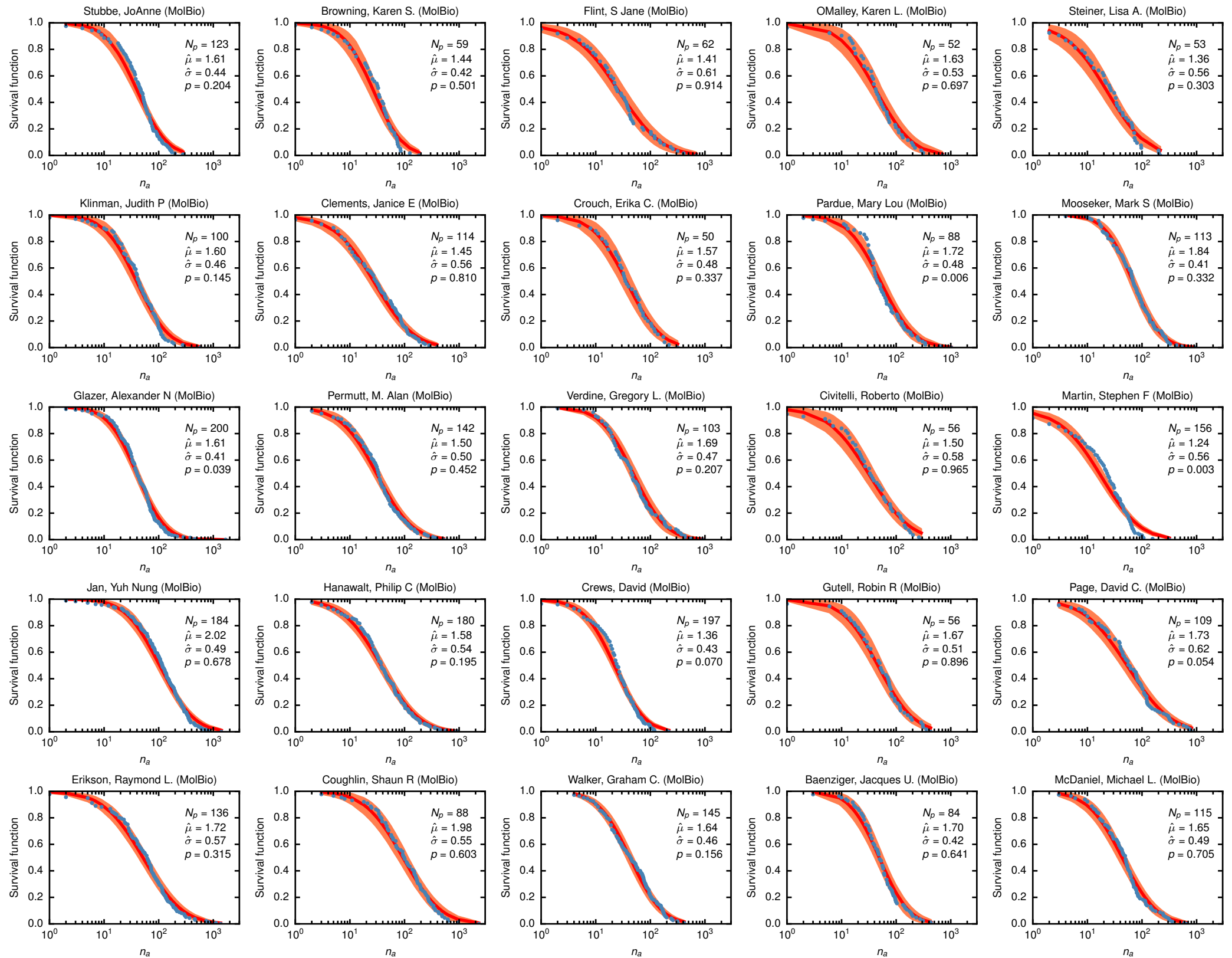

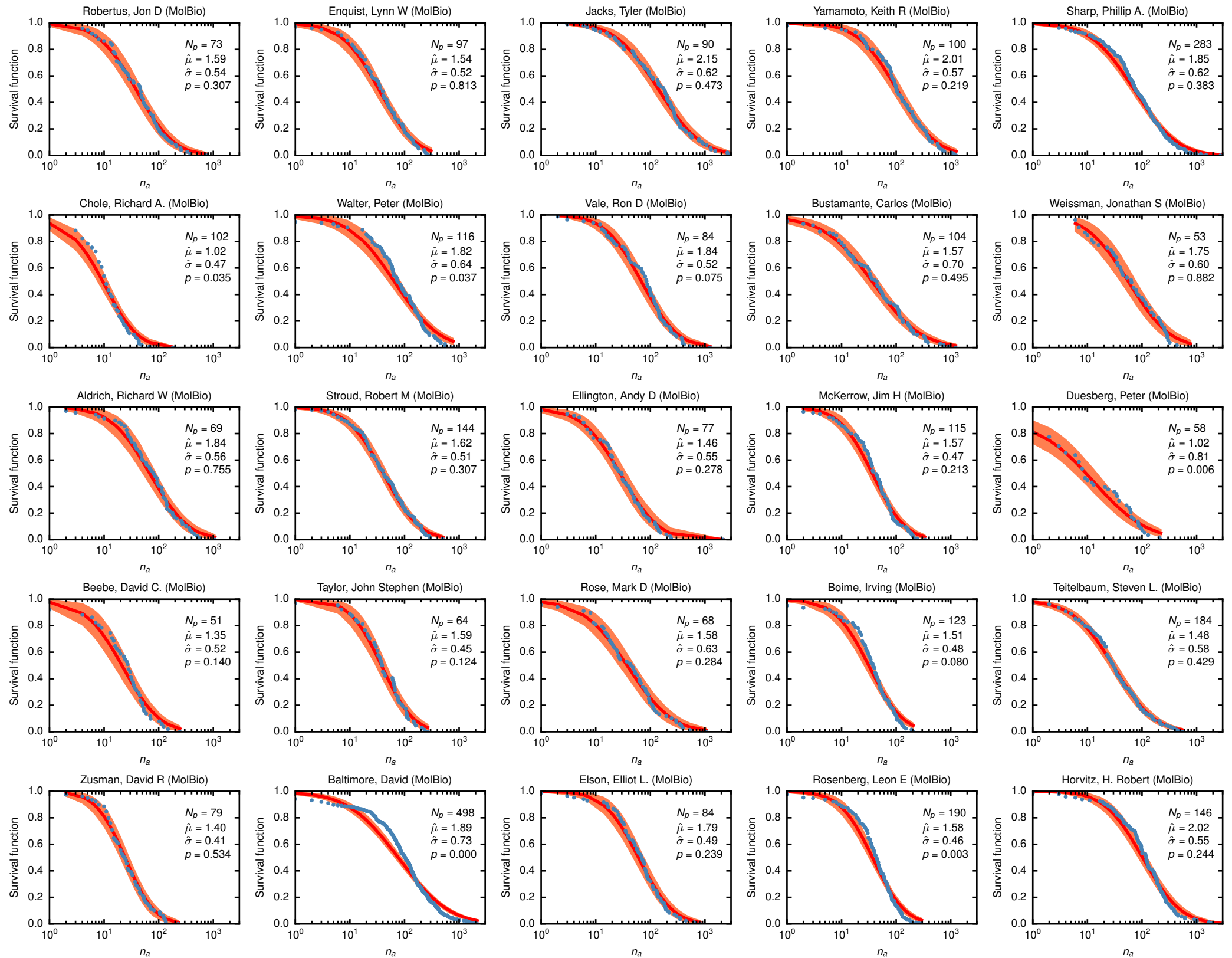

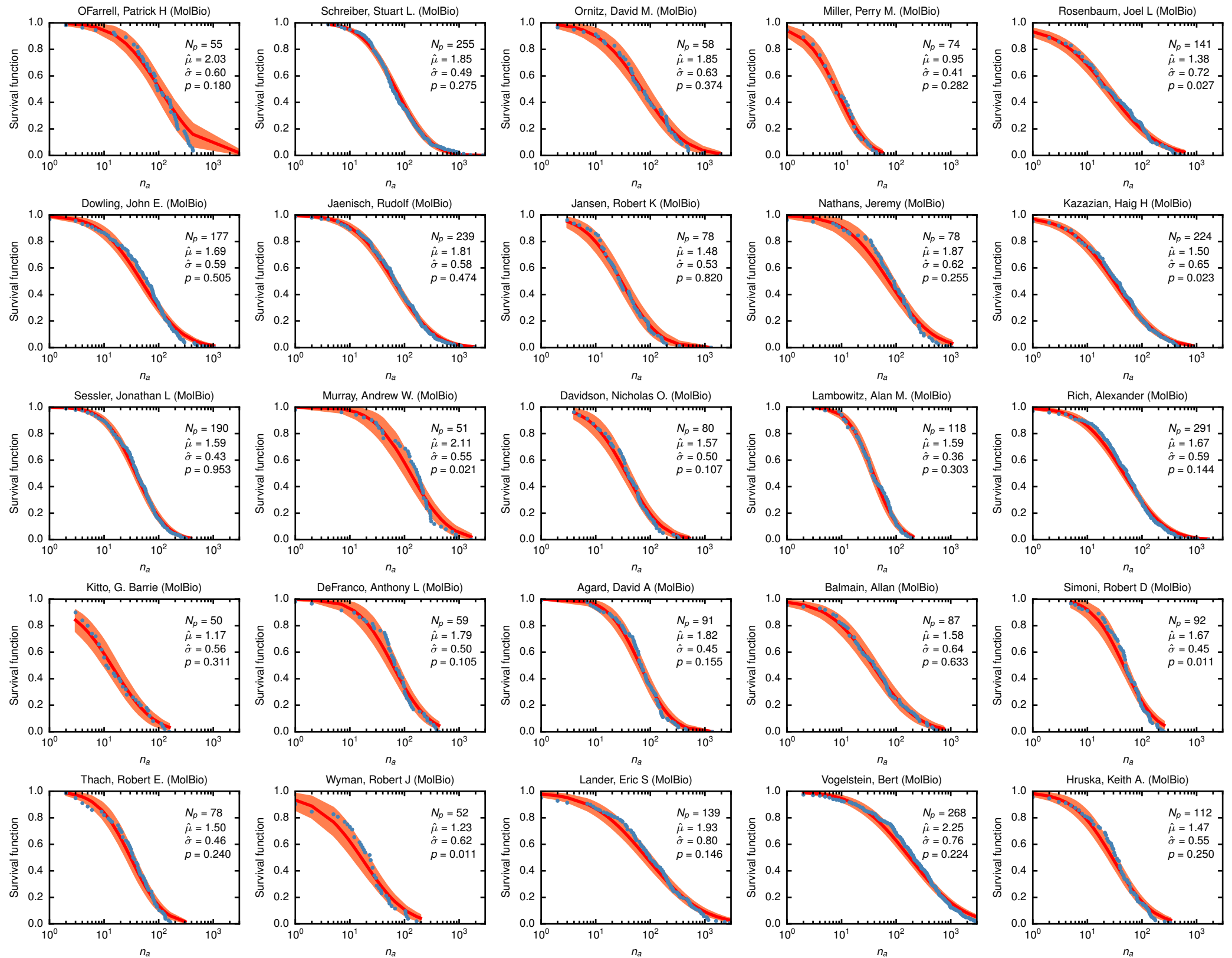

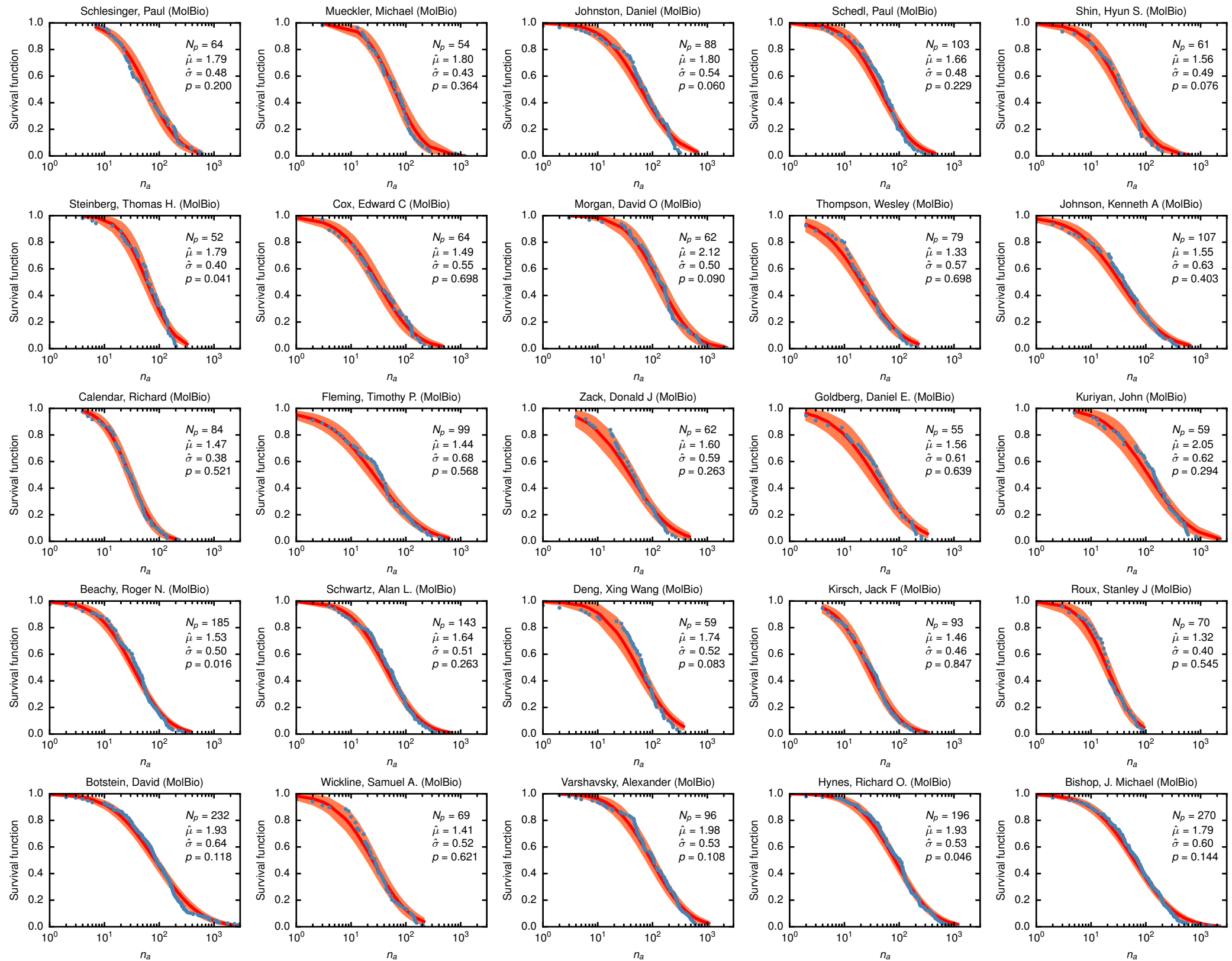

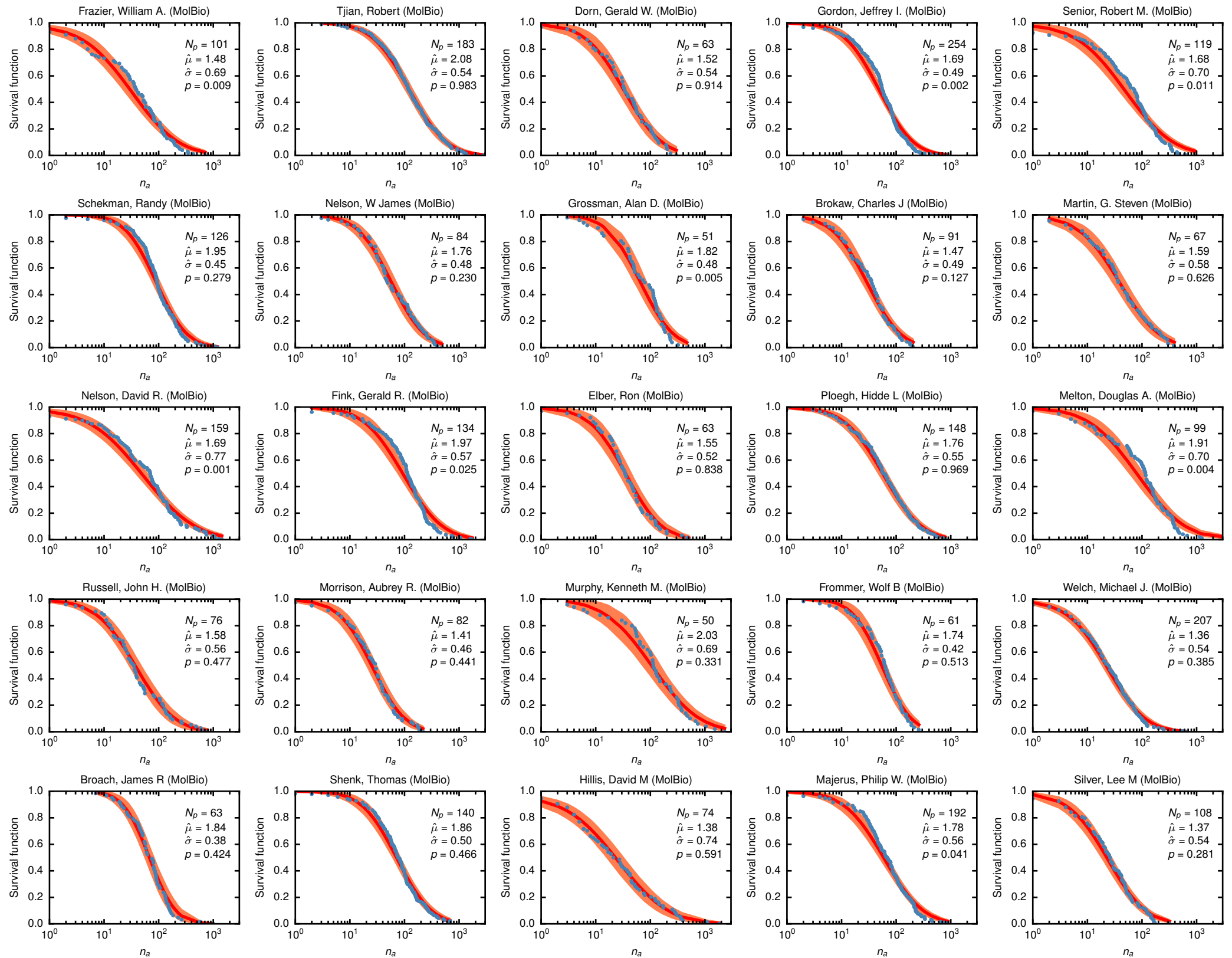

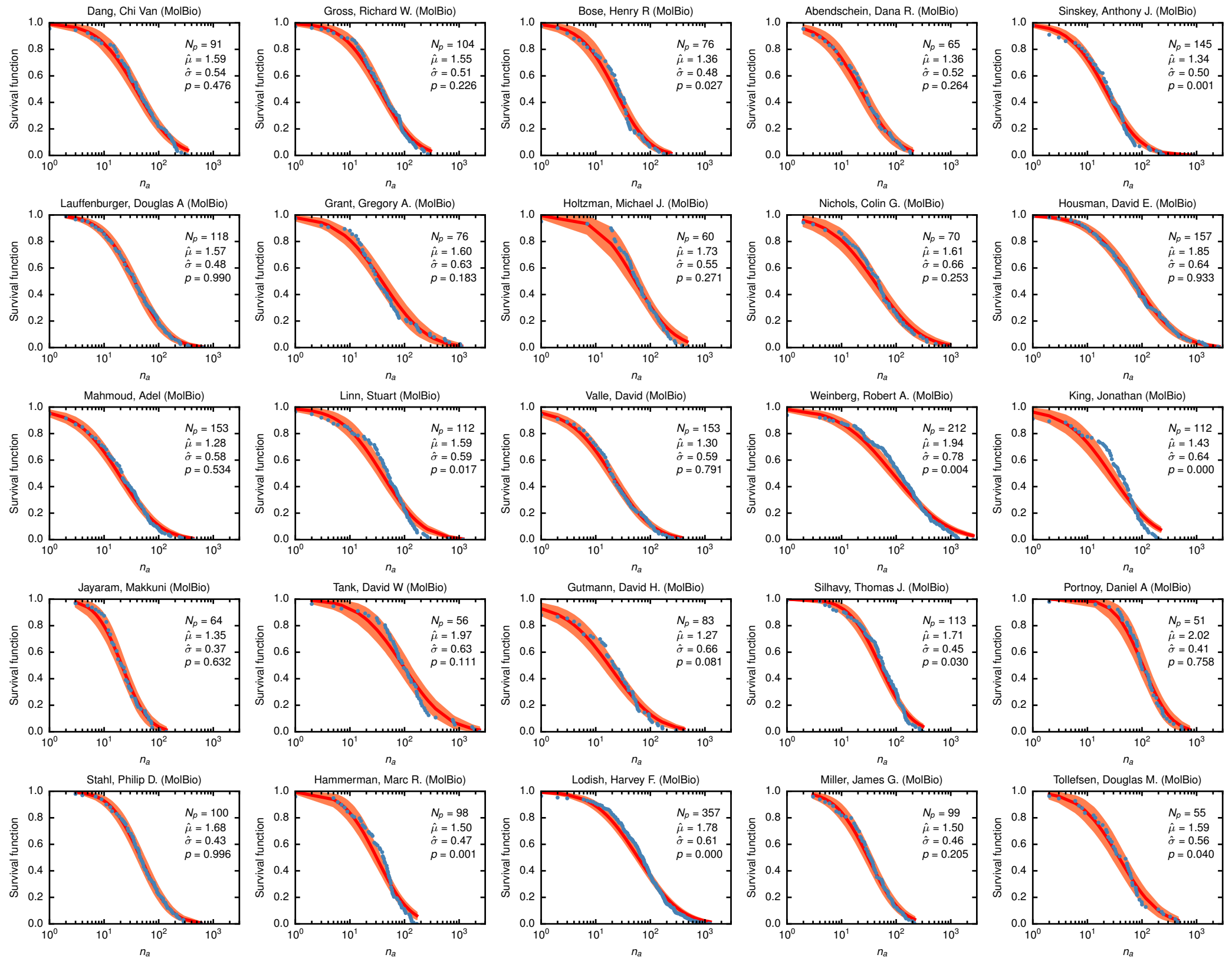

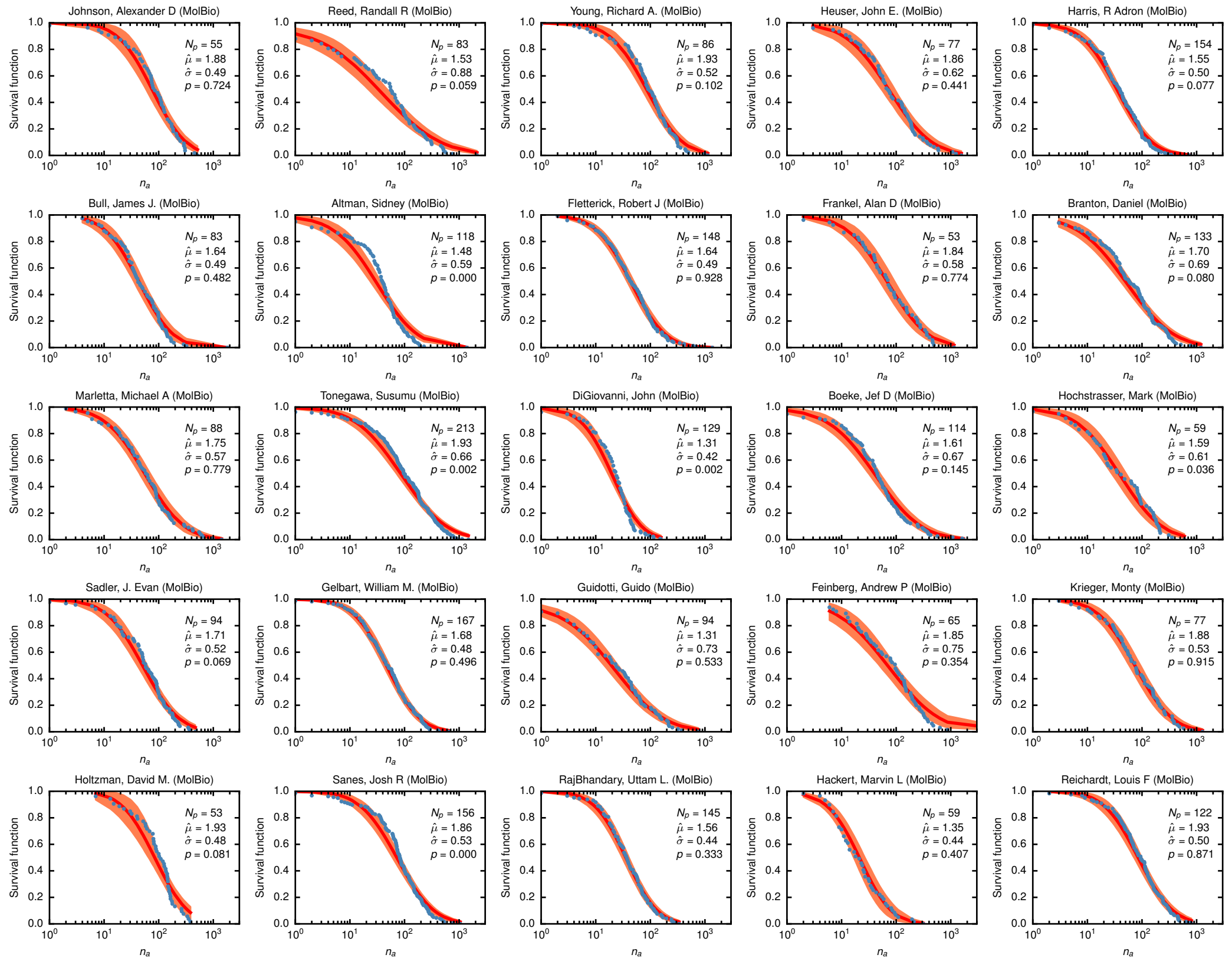

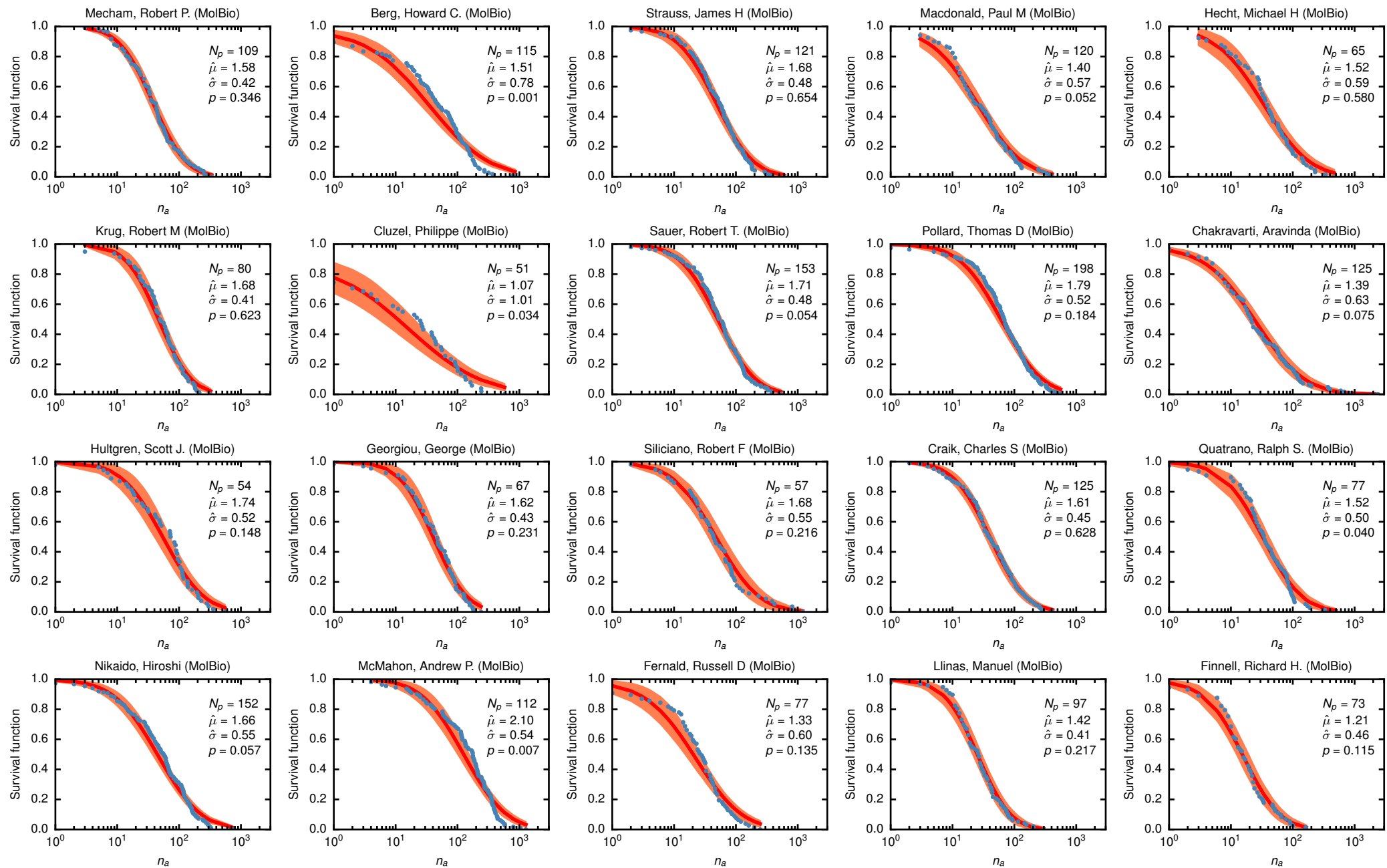

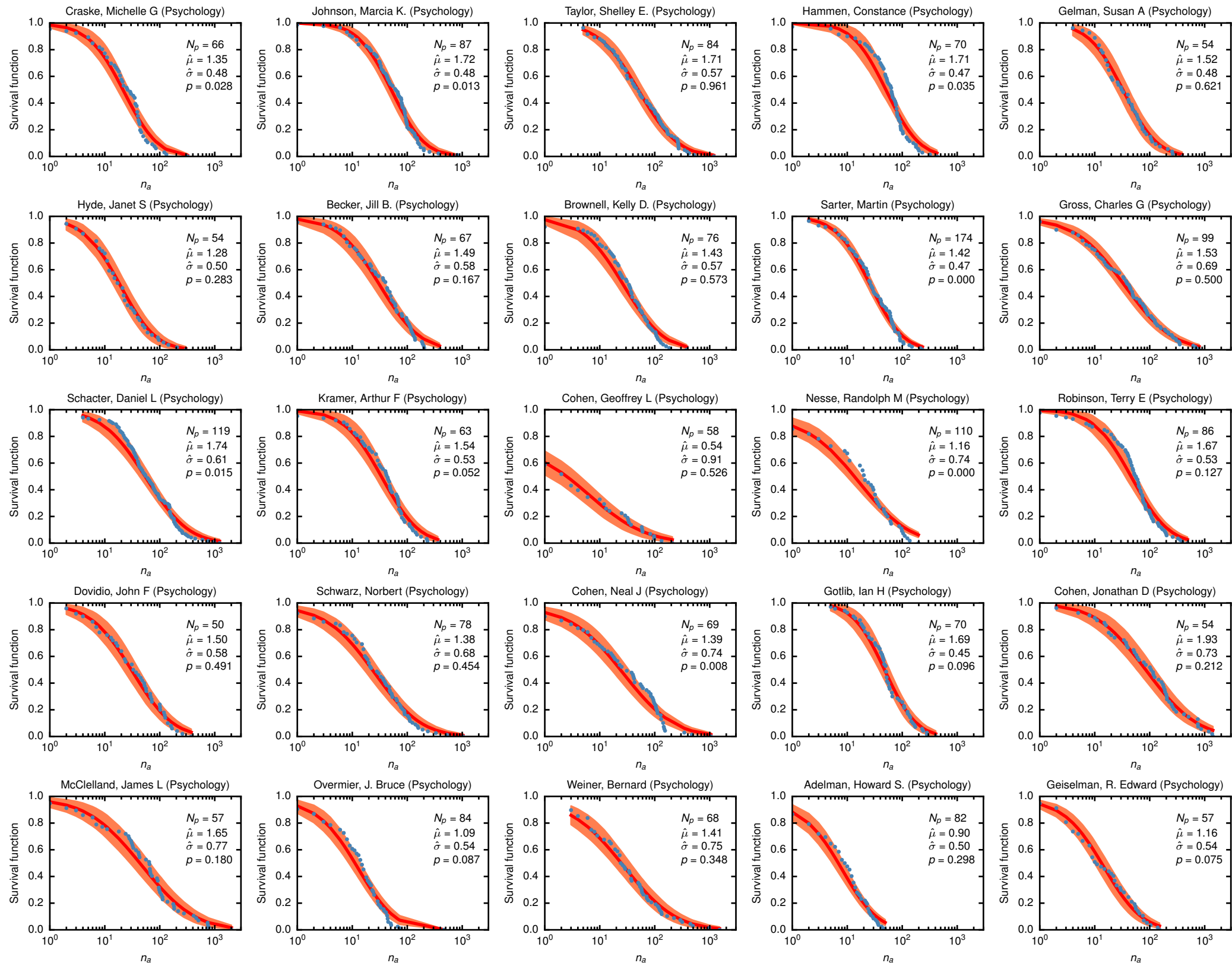

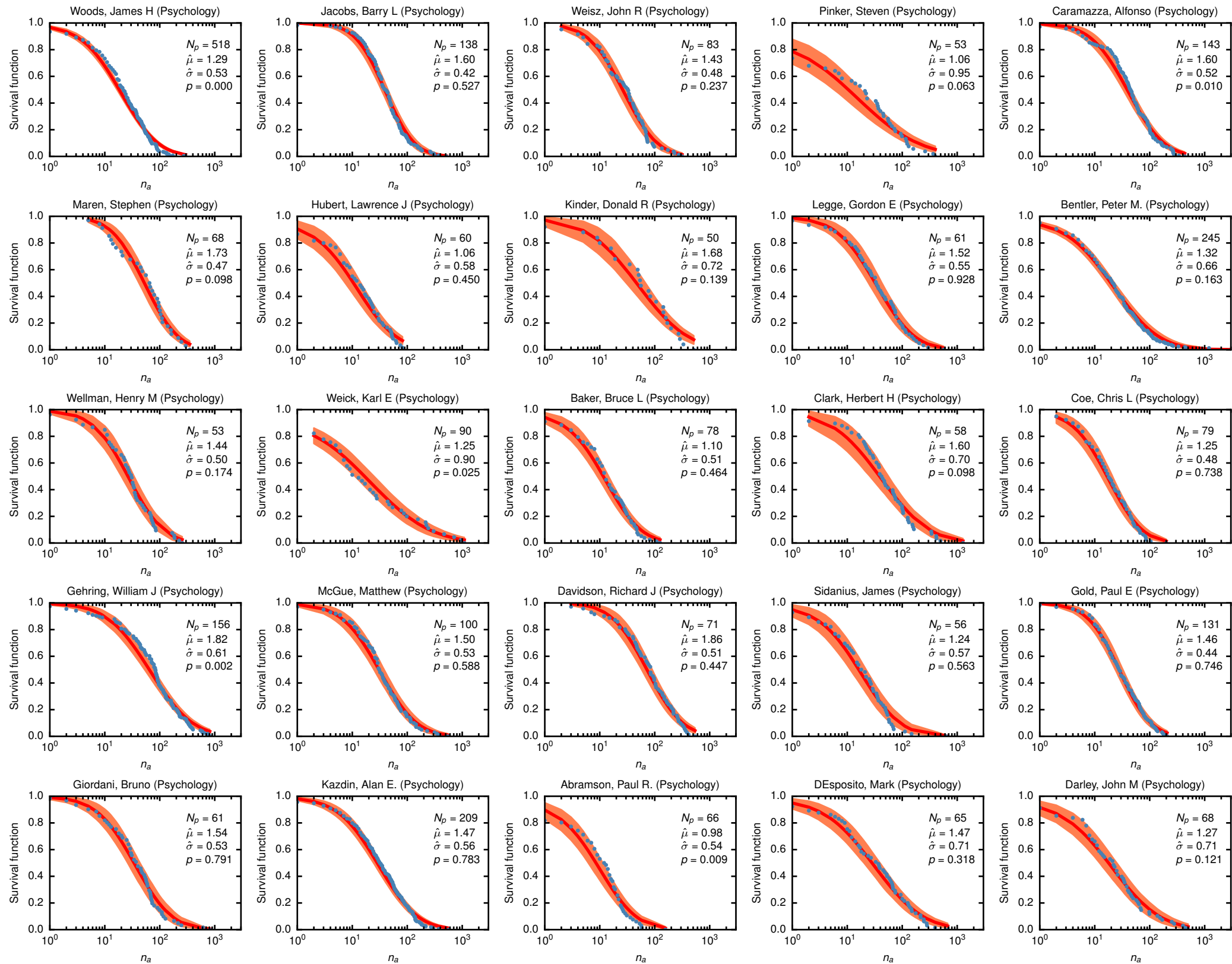

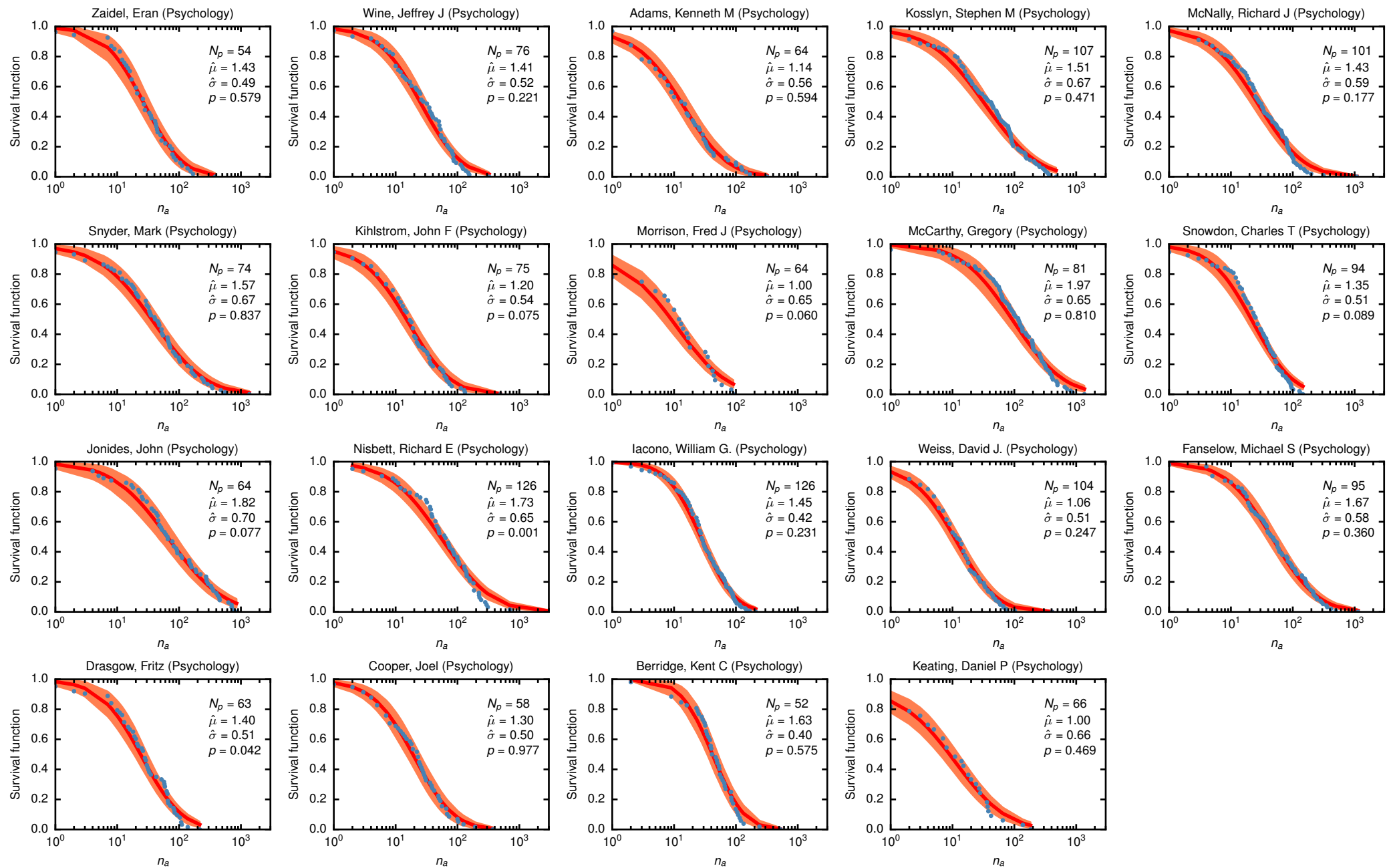

Supplement: S1 File — For a detailed description of the plots see the caption in Fig 1. (PDF) [file pone.0143108.s001.pdf]

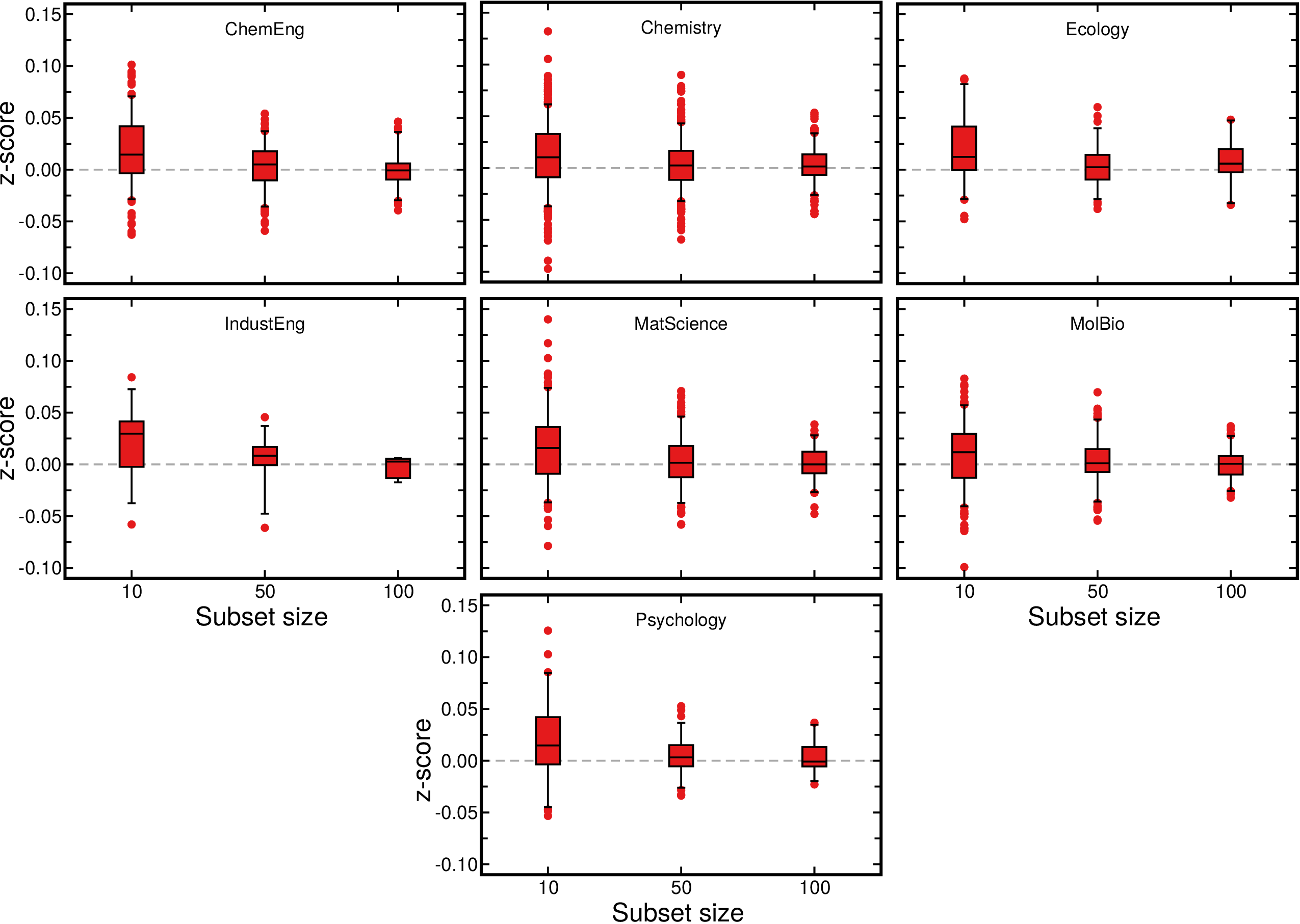

Supplement: S1 Fig — We fit the model to 1,000 randomized subsets of each researcher’s publication list and compare the μ^ obtained from fitting each subset of 10, 50, and 100 publications with the μ^ associated with the complete publication list. Then, for each researcher and subset size, we calculate a z-score using the mean and standard deviation of the “sub-μ^”. For N p≥ 50, the dependence on sample size is negligible for most researchers. Researchers with N p < 100 are omitted from the calculation on the subset of size 100. (TIFF) [file pone.0143108.s002.tiff]

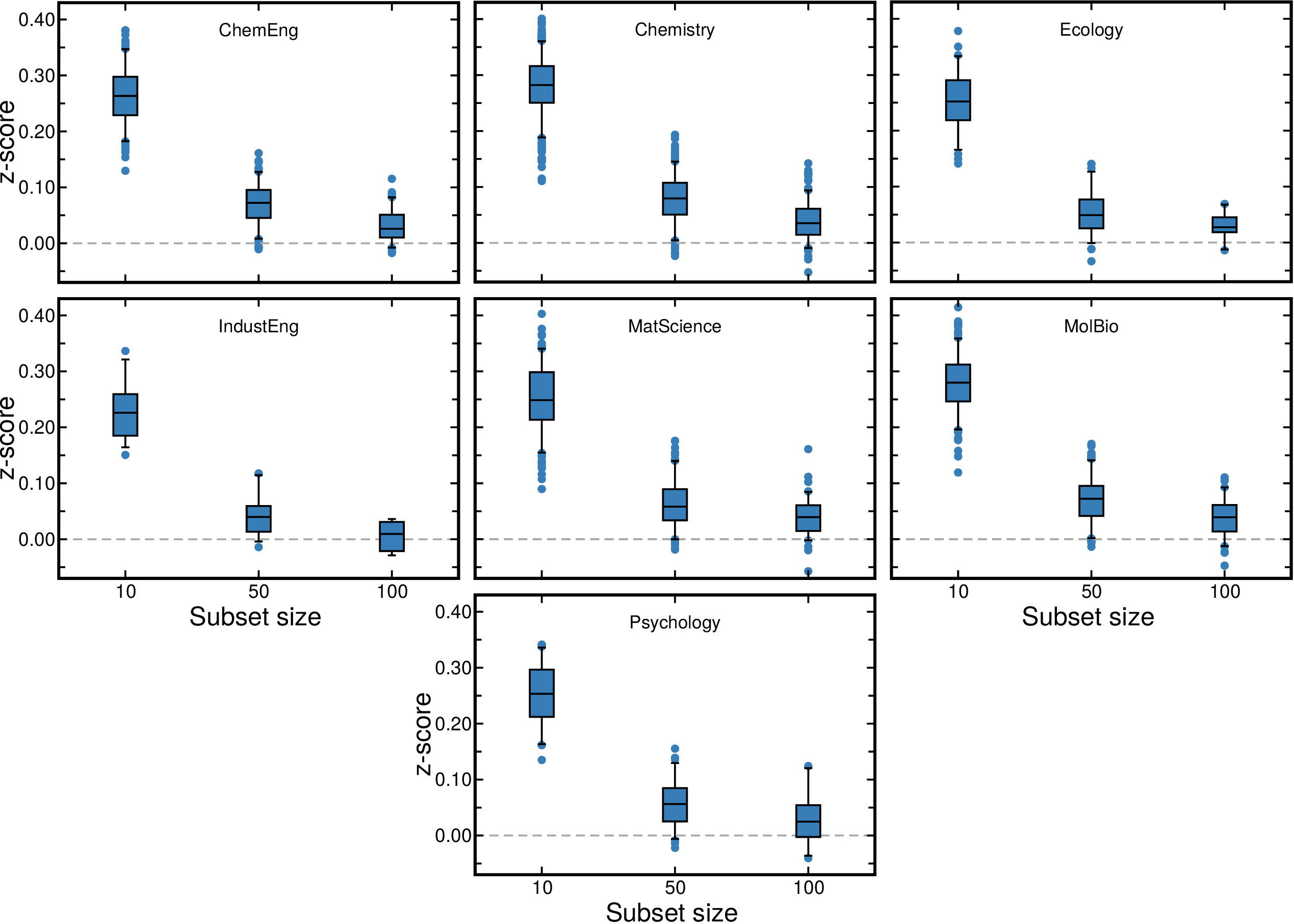

Supplement: S2 Fig — We use the same procedure as in S1 Fig, except here we show the results for the dependence of σ^ on sample size. Estimates of σ^ are more dependent of sample size than μ^. However, as in the case of μ^, the dependence of σ^ on sample size decays rapidly with increasing sample size. Researchers with N p< 100 are omitted from the calculation on the subset of size 100. (TIFF) [file pone.0143108.s003.tiff]

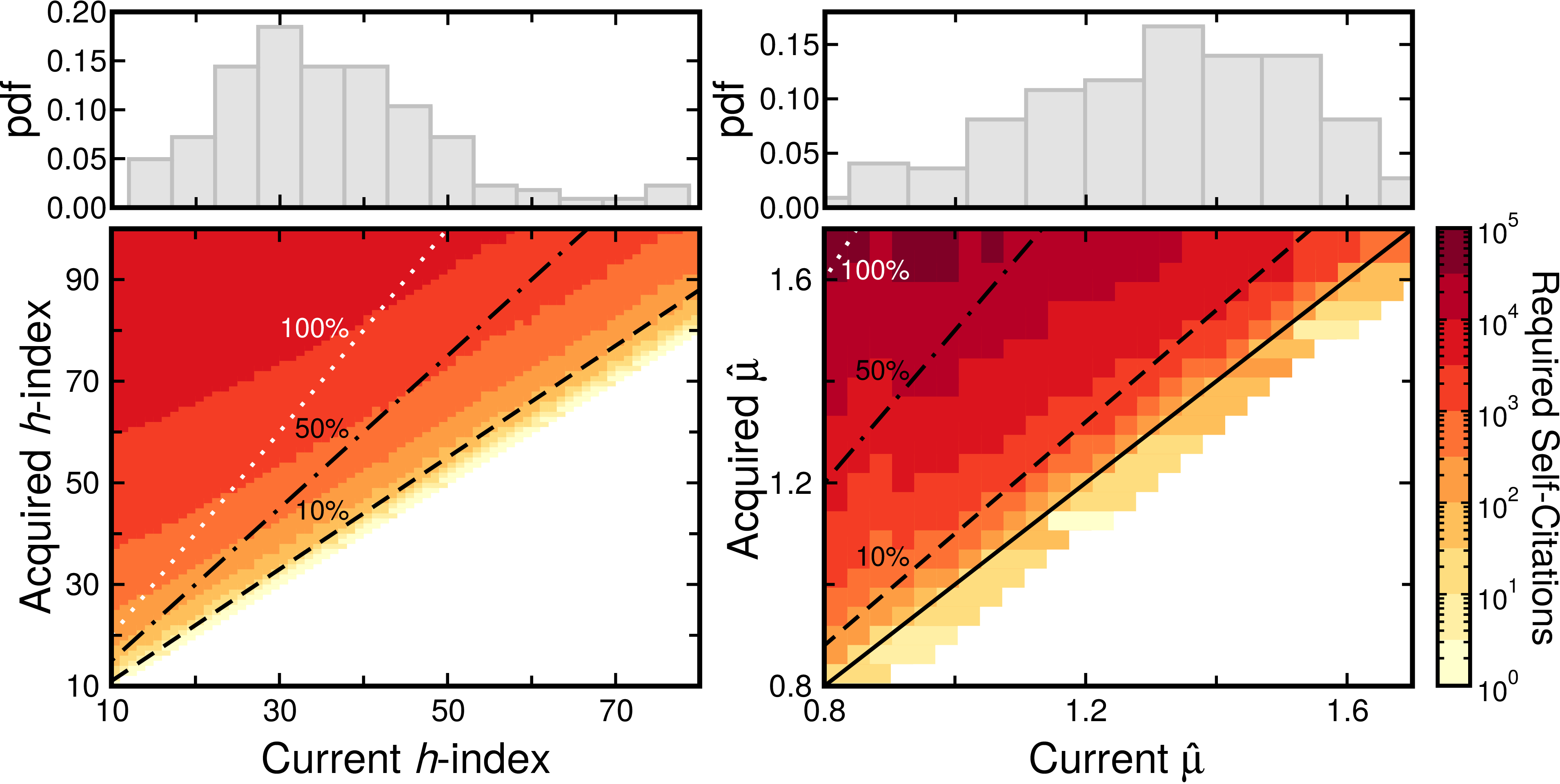

Supplement: S3 Fig — We used the same procedure as in Fig 6, except here we show the required number of publications with self-citations that researchers need to publish in order to increase their indicators. Other details are the same as in Fig 6. (TIFF) [file pone.0143108.s004.tiff]

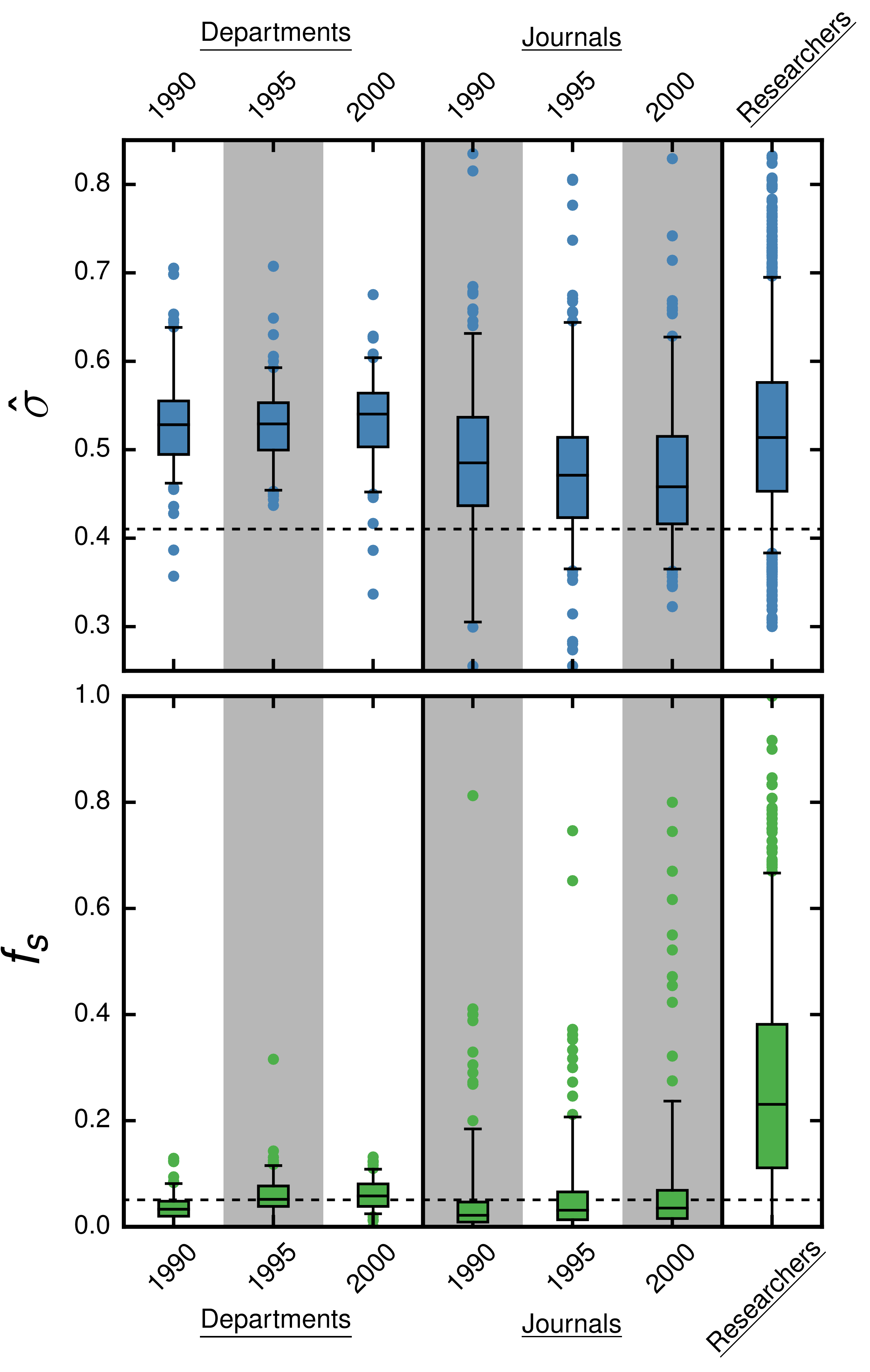

Supplement: S4 Fig — We show the maximum likelihood fitted σ^ (top) and the fraction of secondary publications (bottom) for chemistry departments and chemistry journals in select years, and for all chemistry researchers in our database. The black horizontal dashed lines mark the value of the corresponding parameter for the Journal of the American Chemical Society in 1995. For clarity, we do not show σ^ for 19 journals and 9 researchers that are outliers. (TIFF) [file pone.0143108.s005.tiff]
