# Supplementary material for: The Distribution of the Asymptotic Number of Citations to Sets of Publications by a Researcher or from an Academic Department Are Consistent with a Discrete Lognormal Model
Source: PLoS One. 2015 Nov 16;10(11):e0143108. doi: 10.1371/journal.pone.0143108 (PMC4646658; doi:10.1371/journal.pone.0143108)
Supplement: S1 Table — (PDF) [file pone.0143108.s006.pdf]

S1 Table. Individual lognormal parameters show no dependence on N_p

**Parameter**	**Discipline**	**slope (m)**	**intercept (b)**	$\mathbf{R}^2$	$\boldsymbol{p}$
	ChemEng	0.051	1.218	0.00187	0.5240
	Chemistry	0.073	1.286	0.00668	0.0744
	Ecology	-0.150	1.658	0.00844	0.4502
$\hat{\mu} = mN_p + b$	IndustEng	0.379	0.348	0.04305	0.3166
	MatScience	0.106	1.043	0.01114	0.1278
	MolBio	0.156	1.332	0.01909	0.0410
	Psychology	0.104	1.229	0.00496	0.5732
	ChemEng	0.067	0.379	0.03542	0.0052*
	Chemistry	0.031	0.418	0.00650	0.0862
	Ecology	0.059	0.434	0.00806	0.4524
$\hat{\sigma} = mN_p + b$	IndustEng	-0.094	0.764	0.01657	0.5380
	MatScience	0.033	0.502	0.00921	0.1598
	MolBio	0.064	0.415	0.01688	0.0592
	Psychology	-0.092	0.761	0.02026	0.2302

For each researcher within each of the seven disciplines we perform least-squares linear regression between the lognormal parameters $\hat{\mu}$ and $\hat{\sigma}$, and $\log_{10}(N_p)$. We used a permutation test to calculate the p-values: for each set of pairs, $(\hat{\mu}, N_p)$ and $(\hat{\sigma}, N_p)$, we performed 10,000 random swaps of all N_p and subsequent regression; we obtained a p-value by comparing the original slope of the fit with the distribution of the permuted slopes. $^*p < 0.05/7 \sim 0.0074$.
