# Supplementary material for: The Distribution of the Asymptotic Number of Citations to Sets of Publications by a Researcher or from an Academic Department Are Consistent with a Discrete Lognormal Model
Source: PLoS One. 2015 Nov 16;10(11):e0143108. doi: 10.1371/journal.pone.0143108 (PMC4646658; doi:10.1371/journal.pone.0143108)
Supplement: S2 Table — (PDF) [file pone.0143108.s007.pdf]

S2 Table. Individual discipline statistics of the lognormal model parameters

Parameter	Discipline	Mean	Std Dev	Min	Median	Max
$\hat{\mu}$	ChemEng	1.354	0.256	0.566	1.320	1.921
	Chemistry	1.439	0.225	0.689	1.437	2.179
	Ecology	1.395	0.308	0.702	1.375	1.999
	IndustEng	1.012	0.313	0.603	1.046	1.466
	MatScience	1.250	0.266	0.629	1.254	1.947
	MolBio	1.624	0.253	0.950	1.641	2.250
	Psychology	1.437	0.318	0.545	1.427	1.967
$\hat{\sigma}$	ChemEng	0.508	0.080	0.323	0.513	0.764
	Chemistry	0.468	0.095	0.300	0.482	0.956
	Ecology	0.536	0.124	0.359	0.546	0.896
	IndustEng	0.604	0.150	0.438	0.591	0.796
	MatScience	0.559	0.095	0.335	0.568	0.969
	MolBio	0.525	0.105	0.362	0.541	1.006
	Psychology	0.550	0.137	0.398	0.585	0.954
